# Products in a Pandemic: Liability for Medical Products and the Fight against COVID-19

**DOI:** 10.1017/err.2020.54

**Published:** 2020-05-20

**Authors:** Duncan FAIRGRIEVE, Peter FELDSCHREIBER, Geraint HOWELLS, Marcus PILGERSTORFER

**Affiliations:** * Senior Fellow in Comparative Law, British Institute of International and Comparative Law, London, UK, and Professor of Comparative Law, Université Paris Dauphine PSL, Paris, France; email: d.fairgrieve@BIICL.org.; ** Barrister, 4 New Square Chambers, London, UK; email: P.Feldschreiber@4newsquare.com.; *** Professor of Commercial Law, University of Manchester, Manchester, UK; email: Geraint.Howells@manchester.ac.uk.; **** Barrister, 11KBW Chambers, London, UK; email: Marcus.Pilgerstorfer@11kbw.com.

## Abstract

A multitude of medical products are being developed and produced as part of efforts to tackle COVID-19. They are varied in nature and range from test kits to tracing apps, protective equipment, ventilators, medicines and, of course, vaccines. The design, testing and manufacture of many of these products differs from production in normal times due to the urgency of the situation and the rapid increase in demand created by the pandemic. This article considers the legal issues arising as a result of the production of emergency products, particularly from a products liability perspective. To what extent do existing concepts under the European Product Liability Directive, such as defect, causation and the various defences, permit the pandemic to be taken into account when a Court is considering issues of liability? What is the impact on liability of the modified regulatory regime? In light of that discussion, the case for alternative responses is examined from a comparative and European perspective, including the issue of Government indemnities for the manufacturers of products, legal exemptions from liability and alternative no-fault compensation schemes.

## I. Introduction

The global war against severe acute respiratory syndrome coronavirus-2 (SARS-CoV-2), and the disease it causes (COVID-19), is being fought by many countries on many fronts. Equipment is essential in any war,^1^ and it is no different when that equipment takes the form of medical supplies. Faced with the current pandemic, a vast global demand has arisen for an array of products, from test kits and chemicals, personal protective equipment (PPE), hand sanitisers and other biocidal products, ventilators and similar devices, as well as medicines, treatments and (of course) a vaccine. Producers are now scrambling to meet that demand by ramping up production, developing modified and new products at astonishing speeds, as well as manufacturing in novel ways.

So what of the legal considerations? In this piece, we explore from a legal perspective some of the issues that might arise and how existing legal concepts might respond. Our purpose is not to erect legal road blocks in the way of meeting demand for essential healthcare products; quite the contrary. It is to contribute to the consideration of the application of laws covering product liability and regulation at an early stage, when *ex ante* measures (such as providing warnings, obtaining indemnities from government, etc.) are still available to producers and lawmakers.

## II. SARS-CoV-2 and medical products: an overview

### 1. The virus

The science surrounding the SARS-CoV-2 virus, and the COVID-19 respiratory disease it causes, remains young.^2^ The virus is thought to be zoonotic in nature, but there is yet to be consensus as to the animal source.^3^ Studies of the genomic features of the virus, including whether it is a product of natural selection in an animal host prior to zoonotic transfer or in humans thereafter, are beginning to emerge.^4^ Other projects are ongoing to track the evolution of the pathogen genome as the virus spreads across human populations.^5^ Current genetic sequencing points to SARS-CoV-2 being a betacoronavirus, closely linked to SARS.^6^

The primary modes of transfer of the virus^7^ are respiratory droplets (ie by close contact with a person who is coughing or sneezing, such that infected respiratory droplets come into contact with the mouth, nose or eyes) and contact routes (be that direct contact with an infected person or indirect contact with surfaces or objects used by an infected person). There is also some evidence for airborne and intestinal infection routes, which is reflected in the World Health Organization (WHO) precaution recommendations.^8^ Infection with the virus usually leads, after an incubation period,^9^ to symptoms typically of a fever, cough and shortness of breath.^10^ Other symptoms have included chills, muscle aches/pain, sore throat, conjunctivitis, diarrhoea, new loss of taste or smell, rash on the skin or discolouration of fingers or toes and fatigue.^11^ Wider symptoms have also been reported,^12^ but many cases are asymptomatic.^13^ No antiviral treatment is approved to treat those displaying symptoms of COVID-19. The vast majority of those infected (thought to be about 81%) recover without the need for special treatment, taking (if required) pain relief, cough remedies, rest and fluids. A minority (approximately 14%) develop severe disease requiring hospitalisation and oxygen therapy, and a yet smaller minority (approximately 5%) require intensive care, and perhaps mechanical ventilation, often for severe pneumonia.^14^ Those who are older and have underlying health conditions are thought to be more at risk,^15^ although studies are ongoing as to why some young people with no underlying health conditions have developed severe illness.^16^ Uncertainty remains as to the likely mortality rate – the WHO currently estimates this at 3.4%.^17^

The first human cases emerged from Wuhan, China, in December 2019. Since then, the spread has been fast and global, with other hot-spots emerging in Iran, Italy, Spain, the UK, the USA and, more recently, Russia and South America. On 30 January 2020, the WHO declared the outbreak a Global Public Health Emergency and, on 11 March 2020, a pandemic.^18^ Millions of people have been infected, and hundreds of thousands have died.^19^ In the UK, initial modelling indicated that severe cases of COVID-19 could overwhelm the capabilities of the National Health Service (NHS) and lead to more than 500,000 deaths; and when the same model was applied to the USA, 2.2 million deaths were expected with no measures taken.^20^ Such modelling proceeded from the assumption that R_0_ (the reproduction number, or the number of expected infections for each person with the virus, assuming no pre-existing immunity) was 2.4.^21^ As a result of this and similar modelling exercises,^22^ various interventions were imposed on populations to control the spread of infection. Such measures have ranged from individual isolation to social distancing, school closures and, ultimately, general population “lockdowns”. Later work has suggested (albeit with uncertainty) that these interventions have had a substantial impact on transmission and a depressing effect on the effective reproduction number (R_e_), possibly driving R_e_ below 1 (the threshold leading to reduced numbers of cases).^23^ Further waves of infection are, however, possible upon the lifting of such measures,^24^ but the debate continues as to how other methods short of lockdown can be put in place to contain the spread, such as the detailed tracing of cases seen in South Korea,^25^ and how effective such measures will be.^26^

### 2. The regulatory response

The COVID-19 outbreak poses important regulatory problems for the procurement of appropriate medicinal products and diagnostic medical devices for all in the supply chain of these products. These include the NHS and hospital procurement organisations, Public Health England and the manufacturers of life science products.

In the USA, special legislation contained in the Public Readiness and Emergency Preparedness (PREP) Act^27^ empowers the Food and Drug Administration (FDA) to take steps to accelerate the approval to marketing authorisation and assists in the operation of clinical trials supporting COVID-19-relevant products. However, the European regulatory system, including the Medicines and Healthcare products Regulatory Agency (MHRA) and European Medicines Agency (EMA), has only changed inasmuch as decreasing the administrative and bureaucratic burden of submitting applications for marketing authorisation and clinical development programmes and speeding up the process.^28^ The fundamental objectives of demonstrating clinical efficacy and safety and evaluation of the risk–benefit of new products remain unchanged.

The EMA has set out a comprehensive list of guidelines covering the practical implementation of the Medicines Directive.^29^ The EMA is introducing regulatory administrative changes to speed up the development and approval of COVID-19-targeted medicinal products. These include scientific advice, the PRIME scheme and accelerated assessment together with conditional marketing authorisation procedures. Scientific advice for potential novel coronavirus treatments and vaccines will be free of charge.^30^ The PRIME scheme is for priority innovative medicines. It is effectively a partnership between developers and regulators facilitating the generation of robust data on a medicine’s risk and benefit in terms of clinical efficacy and safety, to enable accelerated assessment.^31^ The EMA is also cooperating with the WHO to help the development of potential COVID-19 treatments by facilitating large-scale clinical trials,^32^ and we review these potential treatments below. In terms of vaccines, the EMA is assisting their development, and two candidates have already entered clinical trials. Furthermore, the EMA is advising on the changes and protocols that may be needed to accelerate the operation and conduct of clinical trials.^33^

In the UK, the MHRA is introducing similar amendments to the administrative procedures governing potential COVID-19 treatments and diagnostic medical devices. The agency is developing a package of “flexibilities” to regulatory guidance in order to support the medicines supply chain and healthcare response to COVID-19.^34^ In particular, as regards clinical trials, remote monitoring and remote access to medical records is allowed, and there is no need to inform the MHRA of a temporary halt and restart of trials. A reduction of subject monitoring visits will not require substantial protocol amendments; safety reporting timelines will not always be met, and there will no requirement to report an increase in protocol deviations as serious breaches.^35^ As regards medicines regulation, the MHRA is implementing priority and expedited assessment for national variations that impact the medicines supply chain, as we shall discuss below. The MHRA is also providing an expedited service for enquiries, processing applications for COVID-19-related diagnostic devices and for device clinical investigations.^36^ For the latter, protocol deviations (eg to ensure reproducibility and specificity for antigen and antibody testing) will not need to be notified. Very importantly, the MHRA has now^37^ issued specifications for continuous positive airway pressure (CPAP) machines and ventilators to ensure a continued supply of these critical medical devices during the COVID-19 crisis. Similar new guidelines for protective clothing have now been issued.

### 3. Medical products

As the foregoing has already revealed, combatting the pandemic and its spread through populations, as well as treating individual cases, requires an array of medical products. As the global reach of the virus has extended, so too has demand for these supplies. In this section, we outline some of the products in question, and how, for the purposes of the discussion that follows, it is convenient to categorise them from a legal perspective.

#### a. Test kits

From an early stage in the pandemic’s progress, the WHO has emphasised the importance of testing^38^ in order to monitor trends in the disease, detect new cases, provide epidemiological information to make risk assessments and to guide the modelling of response measures.^39^ Furthermore, testing individuals enables those key workers in precautionary isolation to know whether they have the disease and should remain isolated, or whether they can return to their important work.^40^

There are essentially two types of testing. First are tests to confirm whether a person has COVID-19 at the time of the test. Generally, these antigen tests consist of nucleic acid amplification tests (NAATs) performed on samples (often a nasopharyngeal specimen taken by swab) that detect the unique sequence of virus RNA, such as real-time reverse transcription polymerase chain reaction (rRT-PCR), with confirmation by nucleic acid sequencing if necessary.^41^ A variety of tests have been developed,^42^ including in the UK.^43^ Testing can deliver a result in a matter of hours, although more rapid antigen tests are also possible.^44^ The second type of test seeks to identify whether a person has had the infection in the past by detecting antibodies that attack the virus (serological tests).

Some difficulties with testing have started to emerge. Shortages of the chemicals required have been reported owing to the unprecedented demand.^45^ Furthermore, some rapid test kits purchased by national governments have proved unreliable: in Spain, kits were reported achieving only 30% accuracy rates,^46^ and similar reports are emerging from other countries.^47^ As a result of such complaints, in order to clear Chinese customs, Chinese exporters must now provide a declaration and evidence that the product is registered in China and that it meets the quality standard requirements of the importing country.^48^ Whilst many manufacturers are now developing or have developed antibody tests that allow individuals to obtain a result in a matter of minutes,^49^ Public Health England currently advises against their use^50^ and their effectiveness continues to be studied.^51^

Pursuant to the In Vitro Diagnostic Medical Devices (IVD) Directive 98/79/EC, test kits that are intended to be used by patients at home need to be assessed by a notified body, in addition to the usual requirements of manufacturers to specify device performance characteristics and self-declare conformity with the safety and performance requirements in the directive.^52^ The MHRA has stated that there are no CE-marked tests for home use, and it is no longer accepting applications to place test kits on the market.^53^ Other European countries have also banned the sale of rapid diagnostic tests due to the risk of misinterpretation of results.^54^ Nonetheless, tests are available on the market, and a working group of Member States’ competent authorities has identified several devices with fraudulent documentation, incomplete technical files and unsubstantiated claims.^55^

#### b. Personal protective equipment

The widespread use of masks to prevent the transmission of SARS-CoV-2 can be seen daily on television news reports. The use of masks was more common in Asia even before the outbreak, but extensive use can now be seen in other parts of the world. Some countries have gone further, and have mandated their use,^56^ although that is not yet the approach of the WHO. Its guidance recommends use by those coughing or sneezing, but only by a healthy person when taking care of someone else with the virus.^57^ Scientific studies are beginning to quantify the extent to which the virus can travel in the air and the ability of a mask to reduce transmission from symptomatic individuals.^58^ On the back of that, some argue that it is time to apply a precautionary principle to the wearing of such equipment.^59^ In addition to masks, other PPE is required by healthcare workers as part of a package of administrative, environmental and engineering controls.^60^ These include visors/goggles, surgical gloves, gowns and aprons and respirators. The UK government has promulgated guidance for PPE for use by health and social care workers in the context of the pandemic.^61^

The rapid spread of the disease has led to an insufficient global stockpile of PPE, with limited capacity to expand PPE production^62^ and the emergence of some protectionist national practices.^63^ Measures are in place to ease the supply.^64^ To assist manufacturers to increase supply, the European Commission has provided a Q&A document concerning conformity assessment procedures for protective equipment, summarising the regulatory standards and requirements that apply to different types of PPE.^65^

As well as regular manufacturers ramping up production, mask design specifications for 3D printing have been made available and have resulted in individuals and small suppliers with access to such printing facilities manufacturing masks to meet demand.^66^ The potential use of such methods has resulted in specific guidance from the European Commission as to the regulatory regime that applies.^67^

#### c. Chemical-based products

As noted above, one of the modes of transmission of the virus is through contact with contaminated persons, surfaces or objects. Recent studies have shown^68^ that coronaviruses can persist on inanimate surfaces such as metal, glass or plastic for up to nine days at room temperature (the period is shorter at higher temperatures). A total of 31.6% of the viral load of an influenza virus can be transferred with a hand contact period of five seconds (lower with a parainfluenza virus), and it has been shown that, on average, a student will touch their face 23 times per hour, with contact mostly being to the skin, followed by the mouth, nose and eyes. Other work considering the stability of SARS-CoV-2 in particular is also being undertaken.^69^ It is therefore unsurprising to find, alongside guidance to populations regularly to wash or sanitise their hands, that the WHO recommends thorough cleaning of environmental surfaces using common hospital-level disinfectants.^70^ In their recent study, Kampf et al found that coronaviruses were efficiently inactivated by surface disinfection procedures with 62–71% ethanol,^71^ 0.5% hydrogen peroxide or 0.1% sodium hypochlorite within one minute of exposure time. Alcohol-based hand rubs based on 80% ethanol or 75% 2-propanol were also found to be very effective.^72^ The precise chemical make-up of the hand rub has also been found to affect its virucidal activity at lower concentrations.^73^

High demand for such products has resulted in manufacturers of other products switching to the production of hand rubs,^74^ or organisations themselves making their own. The European Commission has provided guidance as to the regulatory environment that applies to such products,^75^ and the FDA has promulgated a temporary relaxation in its enforcement policy applicable to alcohol-based hand sanitisers.^76^

#### d. Ventilators, continuous positive airway pressure and related devices

Treatment in hospital of patients suffering from COVID-19 is primarily with oxygen therapy and, in more severe cases, mechanical ventilation.^77^ The demand for such treatments is posing real challenges not only in terms of hospital space,^78^ but also in terms of ensuring a ready supply of oxygen and access to sufficient ventilation devices.^79^ Reports are emerging of shortages in the supply of oxygen and consequent risks associated with demand on hospitals’ vacuum-insulated evaporator (VIE) liquid oxygen storage tanks.^80^ Furthermore, demand for ventilators has skyrocketed as more people are infected and present with more acute symptoms. The production of existing ventilator devices is being scaled up by manufacturers across Europe, sometimes by existing manufacturers alone,^81^ but also in consortium with others (such as the Air Liquide consortium in France^82^). In addition, other machines are being rapidly designed. A Penlon ventilator device is being adapted from other existing ventilator designs by the ChallengeUK consortium.^83^ Furthermore, ventilators are being built from scratch at astonishing speed: Dyson has been developing a completely new model of ventilator (the CoVent) with the Technology Partnership.^84^ Demand for CPAP devices, which provide pressure to keep airways open for those who are able to breathe on their own, has also increased, and new CPAP devices have been developed over similarly short timescales.^85^ 3D printing of accessories for use with respiratory medical devices has also prompted some regulators, such as the Agence Fédérale des Medicaments et des Produits de Santé (AFMPS), to publish specific guidelines.^86^

The European Commission plans to postpone by one year the date of application of the Medical Devices Regulation 2017/745 due to the COVID-19 outbreak.^87^ It has also published guidance on the regulatory regime relevant to medical devices in the COVID-19 context.^88^ Within that guidance, the Commission recognises that the COVID-19 outbreak can be considered a justified circumstance for Member States authorising the placing of individual devices on the market without the completion of conformity assessments where its use is “in the interest of protection of health”.^89^ The guidance goes on to identify factors that national competent authorities will consider when making that decision, including: (1) the degree of criticality of the use of the device for the protection of health; (2) the availability of suitable substitutes; (3) documentation of compliance with a harmonised standard or other specific technical solutions ensuring fulfilment of the applicable essential requirements laid down in the relevant Directive; (4) review of reports of tests performed by competent bodies; and (5) indications from vigilance and/or market surveillance.

In the UK, the approach is to prioritise exemptions by authorising the supply of non-CE-marked devices in the interest of protection of health pursuant to the relevant provisions of the Medical Devices Regulations 2002,^90^ and, as noted above, the Government has further published a specification for rapidly manufactured ventilators.^91^

#### e. Medicines, blood products and vaccines

The WHO currently reports that no pharmaceutical products have yet been shown to be safe and effective for the treatment of COVID-19, but a number of medicines are being identified^92^ and will soon be studied in clinical trials.^93^ For example, the Solidarity trial compares four treatment options to assess efficacy, namely remdesivir^94^ (an Ebola treatment), lopinavir/ritonavir (an HIV treatment), interferon beta-1a (a treatment for multiple sclerosis) and chloroquine and hydroxychloroquine (malaria and rheumatology treatments),^95^ as well as monoclonal antibodies with activity against components of the immune system. Other work has shown that a single dose of ivermectin (an anti-parasitic drug used in head lice treatments) can deactivate the virus within 48 hours in cell culture. Its use as a treatment in humans remains unproven and depends on pre-clinical testing and clinical trials.^96^ In the UK, the National Institute for Health and Care Excellence (NICE) is reviewing its guidance for the diagnosis and treatment of COVID-19, as well as in relation to other medicines in order to address whether they may exacerbate the COVID-19 illness.^97^ The MHRA also has procedures in place for rapid scientific advice, review and approval, aiming to authorise clinical trials within a week^98^ and aiming to be “as flexible and pragmatic as possible with regard to regulatory requirements for clinical trials”.^99^ The FDA has also produced its own clinical trial guidance.^100^

Antimalarial drugs, including those in the Solidarity trial, have been repeatedly promoted by Donald Trump,^101^ despite there being no existing robust clinical evidence base for their use in COVID-19^102^ and their potential side effects.^103^ Such high-profile endorsement has been criticised as risking unsafe drug use beyond ethical off-label provision during an emergency.^104^ The need for researchers not to lose sight of the fact that experimental interventions carry inherent risk to the patient, as well as the need to move quickly to larger collaborative trials, has been emphasised.^105^ As we shall see, there are also liability issues to consider.

The race for a vaccine is underway, with candidates being tested on animals^106^ and humans. The current landscape is set out in a WHO blueprint paper.^107^ Some pharmaceutical companies are working together to develop potential vaccines.^108^ Whilst many have suggested the timetable to a vaccine may be around 18 months, others are more optimistic.^109^ Whatever proves to be correct, many underline the importance of maintaining quality in the process.^110^

Moreover, away from pharmaceuticals, some studies have shown that blood products derived from survivors of COVID-19 might be effective as a treatment for those suffering with the disease.^111^

### 4. From products to issues

The production and distribution of products such as those discussed above,^112^ in the context of a pandemic, gives rise to a number of legal issues that can be conveniently addressed by grouping them into relevant categories.

#### a. Fraudulent and counterfeit products

First, one might identify the deliberately fraudulent or counterfeit products, which sadly appear when the unscrupulous seek to profit out of an emergency. Fraudulent products claiming to cure, treat or prevent COVID-19 are on the rise.^113^ A recent investigation by law enforcement and regulatory authorities from 90 countries revealed 2,000 online advertisements related to COVID-19 with more than 34,000 unlicensed and counterfeit products, advertised as “corona spray”, “coronavirus medicines” or “coronavirus packages” being seized.^114^ By April 2020, the National Cyber Security Centre (NCSC) in the UK had removed more than 2,000 online scams, including 471 fake online shops selling fraudulent coronavirus-related items.^115^ Amazon itself has deleted over a million products for price gouging or falsely advertising effectiveness against coronavirus.^116^ Plainly, there is much room for enforcement action by national authorities, including under the Unfair Commercial Practice Directive.^117^

#### b. Producers ramping up production: manufacturing errors

The next relevant grouping is where existing producers ramp up the production of existing products in order to meet demand. As noted in the foregoing, this has already been seen with manufacturers of chemicals, PPE and medical devices. At first sight, this is simply a case of a producer doing more of the same, but in times of pandemic, the pressure to increase supply quickly can come at a cost. To increase production volumes, some might be forced to use different raw materials where a ready supply of the usual is not available, additional manufacturing/quality control personnel (some of whom might have been rapidly trained and so be less experienced), different techniques for manufacturing or quality control, etc. All of these can potentially lead to the manufacture of products that deviate from the specification, leading to manufacturing defects or “non-standard” products.^118^ Of course, these sorts of errors can arise in the other categories outlined below, but they are perhaps most conveniently dealt with here. Can the circumstances of urgent demand serve as an important relevant circumstance in such a case? If not, ought the law to provide for exculpation in such cases, or ought the law to continue to incentivise production in accordance with the product specification?

#### c. Novel producers and novel production methods

The COVID-19 outbreak has also resulted in new producers making products. This can be producers repurposing production lines to make sought-after products (eg hand gel or ventilators) that they have not previously manufactured. Alternatively, it can be those with access to small-scale novel production facilities (such as 3D printers) using them to produce in order to help meet demand (eg printing PPE). In addition to meeting applicable regulatory requirements, potential liability for products so manufactured should be considered. In some cases, it might be straightforward to identify the producer; in others (such as where a person “prints” a respirator on a 3D printer, using a specification created by a third party downloaded from the Internet), the position is less clear. Are both the printer and the designer liable? Then there is the role of government. Most usually, this will be one of procurement rather than the actual production of product itself.^119^ However, it might be called upon to provide indemnities to producers where insurance is not readily available.

#### d. Newly developed products

Then there are issues presented by the pressure on producers to develop new products. There is, of course, a spectrum of development, from products that might be based (to varying extents) on existing designs, to others that are designed from scratch. Many – such as pharmaceutical treatments and vaccines – will involve technical research and development, all conducted at speed. What should be the regulatory response to such challenges? To what extent can the determination of liability be affected where the pressure to produce is driven by the pandemic? How should the law balance the need to incentivise and quickly develop essential medical products with imposing responsibility for adverse effects as a means to ensure that individuals are not disproportionately burdened by them? What can be learned from previous responses to pandemics, such as H1N1, and experiences across the Atlantic where exemptions from liability are available?

These and other issues are considered below.

## III. Primary liability

Under the Product Liability Directive (PLD),^120^ a producer is liable to compensate a claimant for damage caused by their defective product. The absence of a specific defence for producing products as part of a response to a health emergency,^121^ as we have seen in other jurisdictions, might be thought a cause for concern. Might a producer worry about future liabilities in a way that might stifle the innovation required to develop speedily a vaccine or medicinal response to the coronavirus, or even exercise excessive caution when producing products essential to a response? Similar concerns were raised during the passage of the Consumer Protection Act 1987 (CPA) through the UK Parliament. Lord Denning, for example, was concerned that strict liability ought not to hamper the development of new pharmaceuticals to combat AIDS that were “for the benefit of humanity as a whole”.^122^ In this section, we consider a number of liability issues that the current emergency draws into sharp focus, including whether the defectiveness standard is sufficiently flexible to be sensitive to the production of products in response to a pandemic.

### 1. Producers and products

Liability under the PLD is imposed on manufacturers (producers *strictu sensu*), those who present themselves as producers (*quasi-producers*), as well as those who import into the EU for commercial purposes (*importers*).^123^ Further, liability can arise for *suppliers*, where the producer cannot be identified by the claimant (eg with unlabelled products) and the supplier fails to provide the identity of the producer, EU importer or their own supplier within a reasonable time.^124^ Generally, there will be little difficulty applying these concepts with respect to products put into circulation in response to the pandemic: in the usual case, a producer will be straightforward to identify. That said, and as we have seen, there are reports of less usual methods of production during the pandemic. Consortia made up of a variety of organisations engaged in the production of, say, a ventilator will give rise to the question of who is the correct target for litigation. Where the defect can be attributed to a particular defective component, the producer of that component may be chosen, but under the PLD, the producer of the finished product will be jointly and severally liable to the claimant if the final product is also defective. Consortia are likely to find themselves jointly fulfilling the role of producers and jointly liable as such. Organisations engaging in such activities may wish to agree *inter se* how liabilities that might arise will be distributed in their contractual agreements. As we discuss below, Governments may also step into the role of a producer to ensure speedy supply of a particular product, such as a vaccine.^125^ Where its functional activities constitute those of one of the categories of producer, there is no reason in principle why a Government cannot be liable.^126^ That said, it is perhaps more likely that Government will remain focused on its procurement role.

Others might also find themselves fulfilling the role of a producer during the pandemic. A school might make its own hand gel for use by students and staff, or a hospital may use its 3D printer to make face masks because a ready supply of finished product is not available. In such circumstances, such organisations may well be acting as a producer, just as the hospital in *Veedfald* ^*127*^ was liable as manufacturer of a liquid used for flushing a kidney in preparation for transplant. A defence is available to a producer who can prove “that the product was neither manufactured by him for sale or any form of distribution for economic purpose, nor manufactured or distributed by him in the course of his business”.^128^ The Court of Justice of the European Union (CJEU) has, however, adopted a restrictive interpretation of this and other defences. Thus, in *Veedfald*, the fact that the liquid in question was manufactured by a hospital for a specific medical service for which the public did not pay and that was financed from public funds generated by taxation would not detract from the economic and business character of manufacture.^129^ Organisations such as hospitals, schools and employers^130^ that engage in the manufacture of their own products may therefore wish to consider carefully the potential implications and the scope of their insurance. Thus, if products are produced for an organisation’s own economic purposes, so saving money by not having to purchase from other producers, the defence is unlikely to apply. By contrast, if they can show that they produced without profit, making the product available for free as a societal service, the defence is likely to be available.

Further issues are thrown up by the 3D printing of products used in the pandemic. As we have seen, components/valves for respirators and other PPE such as masks have been designed electronically and, to meet demand, are being “printed” by a multitude of organisations and individuals with access to 3D printing facilities. Such 3D printing, or, more accurately, additive layer manufacturing, involves the use of a computer-aided design (CAD) file. Whilst this can be created by the user of the printer, it is frequently made available by third parties (be they professional or hobbyist) and downloaded to then be used with a printer. The file contains all of the information required for a 3D printer to then produce the physical item using one or more raw materials specified, and depending on the capabilities of the printing system.^131^ As Howells, Twigg-Flesner and Willett observe,^132^ when applying standard PLD principles, potential liability arises for those creating the physical product in their 3D printers, notwithstanding that the reason for any defect might lie in defective instructions embedded in the CAD file, problems with the 3D printer or raw materials used or that the user of the 3D printer might be unaware of such problems. In the context of the pandemic, if PPE products are altruistically produced in this way and donated, there is scope for the Article 7(c) defence to apply. Furthermore, as we shall see, the defectiveness standard is sensitive to the producer using warnings to modify the level of entitled safety expectations in the product, and thus a person printing a product might have some scope to warn that the product has only been manufactured to conform to the instructions on the CAD file that was used. Organisations offering 3D printer services to end users might also argue that they are simply offering a service. There may in any event be avenues of recourse for the user of a 3D printer down the chain of supply on the basis of other theories of liability.

A further related issue that arises is whether the producer of the CAD file can be liable for recoverable damage caused by defects in the contents of the CAD file. This might arise where a person using a piece of PPE has themselves purchased the CAD file and used a 3D printer to manufacture it. Is the CAD file itself a product to which the PLD can apply? In theory, the issue could apply to an array of informational products (leaflets, guidance booklets, training videos, etc.) that might be produced containing defective information about the virus and that, when followed, result in the user sustaining personal injury damage such as contracting the virus. Products covered by the Directive are defined to be “all movables even if incorporated into another movable or into an immovable. ‘Product’ includes electricity”.^133^ There has been debate, but no EU case law, as to whether or not informational products, such as information within a book or map, or software may constitute a product for the purposes of the PLD.^134^ Whilst there is a case for the existing concept of product under the PLD being wide enough to encompass such informational/digital products, there is no definitive answer, and it is to be hoped that the European Commission will take the opportunity of the current review of the PLD to put beyond doubt that digital products are included within the Directive’s scope.^135^

### 2. The defectiveness standard

The central liability-imposing concept under the PLD is that of defect. The claimant establishes defectiveness by proving the product did not meet the standard set by Article 6 PLD, viz. it failed to “provide the safety which a person is entitled to expect, taking all circumstances into account”. Those circumstances include three statutory factors that are marked out for particular^136^ consideration: (1) the presentation of the product; (2) the use to which it could reasonably be expected that the product would be put; and (3) the time when the product was put into circulation. As the CJEU has now established, determining the issue of defectiveness requires an objective assessment by reference to the public at large and the standard of safety a consumer may legitimately expect.^137^ Where a product has an “abnormal potential for damage”, viz. the risks posed by the product having an abnormal, unreasonable character exceeding the normal risks inherent in its use, the product will be defective.^138^ Thus, a finding of defectiveness is a measure of risk; it applies where a product exceeds a threshold level of risk of harm set dependent upon entitled expectations of the public at large.^139^ Central to this exercise is the comparison of the impugned product with the ordinary or normal level of safety that the public is entitled to expect (not absolute safety), as assessed by the Court following consideration of alternative real (or even hypothetical) comparator products.^140^

The application of these principles to assess the defectiveness of products produced in response to the coronavirus pandemic is likely to arise in a number of different ways, some of which are considered below. The issues are, however, linked by a central question^141^: whether the fact of the pandemic can be a legally relevant circumstance to factor into the Court’s objective assessment of entitled safety expectations. Although the pandemic does not provide a defence, as a matter of principle, may the Court take into account that production was to meet a public health emergency, and that therefore the public is entitled to a lower level of safety than would otherwise be the case? If so, how much weight – in the particular circumstances – can this factor be given?

During the PLD’s lifetime, there has been much debate as to which (if any) circumstances are off-limits for a proper defect assessment. There has been little direct consideration by the CJEU, but famously in *A v National Blood Authority*, Burton J held that considering all of the circumstances meant all of the legally relevant circumstances, and that factors should be excluded from consideration where they fell outwith the purpose of the PLD.^142^ Whether the producer was at fault is one circumstance that uncontroversially falls into this category.^143^ More recently in *Wilkes*, the Court adopted a more “flexible approach … including which circumstances are relevant and the weight to be given to each”.^144^ So, what about the public health emergency presented by the pandemic?

#### a. Manufacturing defects: the problem of the “non-standard” product

The first scenario to consider concerns manufacturing defects. The vast increase in demand for certain products has led to many producers drastically increasing the speed and volumes of production. All other things being equal, one would expect greater production volumes to result in a greater number of non-standard products being produced. If the same proportion of a production run is defective and not picked up by quality control systems, increasing volume will most likely result in a greater number of products with manufacturing defects being put into circulation. Traditionally, liability has readily been found for manufacturing defects, and one might anticipate no change in the attribution of liability just because total numbers increase.

However, other things are unlikely to be equal. As outlined above, producers may be forced to change methodology, raw materials, personnel or approaches to quality control in order to meet demand. Whilst it might be that some changes have the happy side effect of improving the safety of the resultant product, it is perhaps more likely that hastily made changes to production in order to meet demand will increase defect numbers.

Can the fact that more product is being produced in response to the pandemic be a relevant circumstance? We consider this unlikely. “Non-standard” products (viz. those that deviate from the producer’s intentions) will inevitably be compared with the “standard product” (those that do not so deviate).^145^ Where that comparison reveals that it fails to offer the “normal” level of safety that the public – as objectively assessed by the Court – is entitled to expect, then one would expect liability to be imposed. The fact that the pandemic has caused the producer to increase production does not change this. The debate here has distinct resonance with whether the avoidability of the defect or the practicability or cost of precautionary measures should be considered. In non-standard product defect cases, such features have been held to be irrelevant,^146^ a conclusion broadly accepted in later analyses.^147^ This conclusion is, we would suggest, justifiable by reference to the economics. Increased production ought in general to mean increased profits for the producer, particularly in an environment of high demand. Liability for “lemons” deviating from the norm is justifiable by reference to the ability of the producer to spread the risk through price or insurance across the greater volume produced,^148^ by the preventative effect that results by placing liability on the person best able to prevent the damage at the lowest cost^149^ and the particular health interests at stake.^150^

#### b. Comparisons, the group defect doctrine and the time at which the product was put into circulation

In the case just considered, defectiveness is considered primarily by reference to the comparison between the index product and the standard as comparator. Both the deviant and the “standard” product are manufactured at the same time in response to the pandemic. But what about comparisons with products put into circulation prior to the pandemic? Are such comparisons legitimate? Health emergencies aside, one would normally expect the passage of time to increase the level of safety of products that can legitimately be expected. That is why one of the statutory factors that is taken into account by the Court is the time at which the product was put into circulation: Article 6(1)(c). Article 6(2) also speaks to that issue by mandating that “[a] product shall not be considered defective for the sole reason that a better product is subsequently put into circulation”. The present health emergency has the potential to give rise to reverse comparisons, where a product produced in response to the pandemic is worse in terms of safety than a forerunner because of the circumstances of its manufacture or development. An example might be a ventilator adapted at speed from an existing design in response to urgent demand, but that turns out to be less safe than a previous model. As a matter of principle, and on standard principles, there would seem to be no reason to rule out backwards comparison with safer forerunner products. That would also apply where reliance is placed on the group defect doctrine developed by the CJEU in *Boston Scientific*. There, the Court held that a product could be found defective as a result of its membership of a group of products that had a higher-than-normal risk of failure, without the claimant needing to establish that the particular problem arose in the index product.^151^ Ascertaining the normal risk in such cases is often an exercise in backwards comparison, looking to previous groups, such as batches or production series. Groupings defined by reference to production methods during the pandemic will engage with such principles.

That said, as noted above, one of the particular factors identified in the PLD is the time when the product was put into circulation: Article 6(1)(c). That wording, it seems to us, is wide enough not only to require the orientation of entitled safety levels to a place on a temporal scale of product development and safety enhancement, but also by reference to other temporal factors insofar as they impact (perhaps even temporarily) on the legitimate safety expectations of the public at large. Thus, during periods of wartime, additional or different risks may arise that, in turn, might cause the entitled safety expectations of particular products to increase or decrease. This is likely to depend on the nature of the product and the relevant risks that apply to its foreseeable use. Another example might be where, during a particular time period, a relevant risk that might otherwise be faced when using a product is limited or extinguished in some way. Thus, the legitimate expectations as to safety in respect of clothing foreseeably to be worn in jungle regions where there is a high risk of a deadly mosquito-transmitted infection are likely to extend to such products providing protection to reduce that risk. If the risk of contracting such a disease is drastically reduced to near zero through anti-mosquito measures, then for products put into circulation subsequently, the entitled safety expectations in respect of the clothing may reduce. That can in theory remain the case even if (say, after a decade) the disease makes a comeback and entitled safety expectations change again.

It seems to us that, in the same way, entitled safety expectations may in theory be modified during a period of a health emergency. However, we would emphasise two important points. First, the modification will not always be such as to lower entitled expectations; indeed, a court would be able to conclude that, on an objective assessment, legitimate expectations as to a particular aspect of safety have increased (eg where foreseeable use of the product gives rise to a risk of infection of what is a novel virus). Secondly, the court will need to distinguish carefully between temporal factors that impact on entitled safety expectations (which can be taken into account under Article 6(1)(c)) and those that merely impact a producer’s ability to manufacture in accordance with such expectations (which, as we have explained above, are unlikely to be relevant). Within these limits, Article 6(1)(c) is likely to provide some flexibility to argue that the public’s entitled level of safety expectation has been affected.

#### c. Benefits and social utility

A further way in which the pandemic might be relevant to the assessment of defectiveness concerns whether, and the extent to which, the benefits of a product may be weighed against its risks when considering whether it is defective. The benefits of a SARS-CoV-2 vaccine, COVID-19 treatment or ventilator may be profound in the context of a health emergency. If legally relevant, they might weigh so as to lower the entitled level of safety expectation in respect of risks, including even serious side effects. A related issue, considered in Section V below, is whether regulatory approval is relevant, particularly where the processes of scrutiny or standards applied have altered in response to the health emergency.

Early English cases ruled, as a matter of principle, that a product’s benefits should be left out of account, by reference to the *traveaux préparatoires* ^152^ and purpose of the Directive.^153^ Later cases have reached the opposite result, albeit making a distinction as to relevance in non-standard and standard product cases.^154^ Other European jurisdictions, such as Germany, have embraced considerations of risk/benefit more enthusiastically.^155^ At the European level, the CJEU has not directly addressed the question, although it is perhaps notable in *Boston Scientific* that defect was assessed without any reference to the benefits or utility of the product. Furthermore, in *W v Sanofi*, the Advocate General rejected a submission that a broad assessment of cost/benefit was required when considering a vaccine,^156^ which of course provides benefits both to the user and society more generally through herd immunity. However, the Advocate General’s opinion was thought in the English case of *Gee* to be directed at a particular submission in *W*, and only to suggest that a balance of costs and benefits was not *necessarily* required.^157^ In the course of her discussion, Andrews J posited the example of a new chemotherapy drug that had proven advantages over others on the market, but also had a rare and serious side effect. She reasoned that it would be difficult to see how the level of entitled safety expectation could be evaluated without taking into account the products particular benefits – namely the enhanced efficacy.^158^ Yet this could be done by considering in particular the presentation of the product: the presence or absence of a warning of a rare but serious side effect (either a known risk or a discoverable one^159^) could well provide the answer without recourse to arguments about efficacy or other product benefits. Indeed, it might be thought odd were the PLD to avoid a defect finding by reference to the benefits of a product where a known (or knowable) risk of harm was kept from the consumer, thereby depriving them of an effective choice on running that risk. Other difficulties may arise in cases where the product’s benefits might vary. Any vaccine that is developed in response to the virus might initially be effective, but later become less so with subsequent virus mutation. It is fair to say that the issue remains controversial legally, and ripe for further argument in the context of an emergency.

#### d. The role of warnings

Perhaps the most obvious way in which legitimate safety expectations may be lowered in the context of the pandemic is by a producer providing information and warnings accompanying the product as part of its presentation. This must be considered by the Court pursuant to Article 6(1)(a) PLD.^160^ A product that poses a known or knowable additional risk because of production at a time of pandemic, but that warns about that additional risk in its presentation, allows a consumer autonomy of choice and self-determination. Even if such risks are not posed by comparator products, including those developed earlier in time, the entitled safety expectations as judged by the court are likely to be sensitive to such presentation. A producer who provides a warning that a ventilator has not been subject to a particular material test because it has been rapidly adapted from an earlier design, or the producer of a medicine who draws the prevalence and seriousness of the risk of a particular side effect to the attention of the user, may succeed in altering the Court’s assessment of the level of safety that the public is entitled to expect from it.

A related issue is the relevance of the fact that many medical products will be provided to a user through a doctor or other learned intermediary. In England, the relevance of this factor has been controversial. In *A v National Blood*, Burton J refused to transfer knowledge of a risk possessed by the intermediary to the patient.^161^ Later cases, however, have shown more flexibility: in *Wilkes*, the Court (proceeding by agreement) regarded the presence of an intervening healthcare professional as a relevant circumstance.^162^

From this discussion, it follows that the more specific the warning and the more likely the warning is to reach the ultimate user of the product, the greater the chance of the producer avoiding liability. We see this as a particularly useful tool for a producer to alter legitimate safety expectations in the context of this pandemic.

### 3. Causation

It is only for “damage *caused* by a defect in his product”^163^ that a producer will be liable. Whether damage is so caused, and the extent of any such damage, must be proved by the claimant.^164^ Subject to EU principles of effectiveness and equivalence, national rules of causation are applicable in claims brought under the PLD.^165^ This will result in a claimant’s prospects of success on this issue varying depending on the jurisdiction. In *W v Sanofi*, the CJEU gave some guidance in a case where a patient developed deteriorating symptoms of multiple sclerosis shortly after he was vaccinated against hepatitis B. The Court held that the burden of establishing causation would be excessively difficult for a consumer to discharge if national law prohibited recourse to circumstantial methods of proof and instead required proof through scientific or medical research.^166^ Conversely, national rules were not allowed to make it too easy for the claimant, and thus evidence must be “sufficiently serious, specific and consistent” to establish the defect as the most plausible explanation for the damage.^167^

In addition to scrutinising applicable national rules against such principles, claims arising in the context of the pandemic may give rise to a number of particular causation issues.

First, there are those cases where the use of the product is said to have caused a particular side effect, such as in *W v Sanofi*. This type of issue is perhaps most likely to arise where the product is a medicine or vaccine, but it could also be an issue where a device or piece of PPE is said to have caused an adverse reaction. Much here will depend upon the content of national rules and the precise factual picture concerning the side effect in question and its mechanism, prevalence and link with the product both generically and individually in the particular case. Where even a non-defective product would carry the risk of developing a particular adverse effect, the Court may need to engage with controversies such as how “defect” is to be conceptualised^168^ and whether it is necessary for the claimant to establish that the relative risk of the adverse event with the defective product was greater than 2 in order to succeed in the causal enquiry.^169^

A second type of case is where it is alleged that the defect in the product caused the claimant to become infected with the virus and thereby sustain recoverable damage. A defect in a piece of PPE, or in the composition of a hand gel, or even in a vaccine, might result in that product providing inadequate protection against infection by SARS-CoV-2. In such a case, if a claimant becomes infected, causation issues will arise as to: (1) whether the claimant became infected as a result of the defect in the product, as opposed to some other source; and (2) whether even if s/he did, s/he would have been likely to be infected and sustain the same or more serious damage in any event. The burden in each case is on the claimant. In England, where there are competing potential causes, the judge must be satisfied that the defect in the product was the more probable. That may be straightforward enough where the competing explanations are themselves not improbable (even if uncommon). However, the judge is not compelled to accept a remaining improbable theory after having eliminated impossible theories from those advanced by the parties.^170^ Applying such an approach to issue (1), causation may prove very difficult to discharge. Whilst there is much still to learn about this particular virus and its mechanism of transmission, current indications are that it is highly transmissible and infectious.^171^ This, coupled with both an incubation period and increasing general prevalence throughout populations, is likely to make the claimant’s task of proving the defective product as the reason for the infection more difficult. These features are also likely to pose challenges where issue (2) arises: the more other opportunities for infection, the more likely it will be that infection and damage would have been sustained in any event. Even aside from infection, there might be arguments as to whether the ultimate patient outcome would have resulted in any event. Would a patient who became infected due to a defective product have died anyway within a similar timescale? Some work suggests that although the death rate is higher than previous averages for the time of year, given the profile of those most at risk, a considerable proportion of the deaths might have occurred in any event.^172^ Does it matter for causation purposes whether, factually, there would not have been a non-defective alternative product available to the claimant (eg a non-defective ventilator instead of the rapidly produced defective one that was used)?

Other issues may also arise in relation to the chain of causation. Defective tests for either the virus, or for antibodies to it, may give false-positive or false-negative results. A false-positive result that a person has the virus at a point in time is less likely to result in personal injury being sustained, but harm resulting from a false-negative result may extend beyond damage to the patient in question (whose treatment might be delayed^173^) and lead the person in question to engage in activities (or not triggering contact tracing) such that others become infected. Quite apart from the real practical issues about establishing the true cause of infection in a particular case, quite when the chain of causation can be said to be broken might prove important. Similarly, with defective antibody tests, a false-negative result (that someone has not had the virus) might lead to over-caution and perhaps pure economic losses (not recoverable under the PLD), but probably not personal injury. False-positive results might not only result in the patient exposing themselves to the risk of infection in a way that they would not have otherwise done,^174^ but also their increased exposure might lead to infection and them infecting others unknowingly.

## IV. Development risks defence

Article 7(e) PLD provides the producer with a defence if he proves “that the state of scientific and technical knowledge at the time when he put the product into circulation was not such as to enable the existence of the defect to be discovered”.

This provides an exculpatory route for the producer when the defect could not have been known about. It is most likely to arise in relation to the current pandemic when new products are being created, such as vaccines, medicines and test kits. For existing products that are being adapted or used to treat COVID-19, it would only be relevant if the virus created a danger by interacting with those products in a manner that could not have been anticipated. The question addressed here is not so much about gaps in knowledge related to the virus and COVID-19 (of which we know there are many), but rather about the lack of knowledge of risks posed by the product. Where the risk emanates from the interaction between the product and the virus/COVID-19, there will be issues about the extent to which knowledge was discoverable based on extrapolations from the existing state of scientific and technical knowledge.

The development risks defence was one of the most controversial aspects of the PLD. It was not included in the original text and was forced upon the Commission by Member States concerned to protect innovative industry. The reluctance of the Commission to embrace the defence is reflected in it having being made optional.^175^ The Commission’s lack of enthusiasm for the defence also suggests that it was intended to have a narrow scope, although its actual breadth will have to be derived from the text of the Directive as interpreted by the Court of Justice. The burden is squarely placed on the producer to establish the defence and further suggests a narrow role.^176^ The parameters of the defence define just how strict the product’s liability regime is.^177^ However, even if the defence is narrowly construed, that does not mean that the regime is oblivious to the challenge faced by those producing goods to meet emergencies. Our discussion of defect illustrated how many of the concerns of producers about unfair expectations of safety when responding to an emergency can be assuaged by taking into account the time at which the product was put into circulation, or pragmatically adjusting entitled expectations by reference to warnings as to risk given by the producer. This defence is about unknown and unknowable risks, not about known risks in an uncertain environment.

In exploring the contours of the defence available under the PLD, guidance can be found in the decision of the Court of Justice in *Commission v United Kingdom*.^178^ The Commission considered that the UK’s implementation of the defence was too generous. The UK’s implementation determines knowledge by reference by the standards of “the producer of products of the same description as the product in question” and talks in terms of whether such a producer ”might be expected to discover the defect”, thus potentially introducing expectations into a test that seemed, in the PLD, to be based on simple discoverability.^179^ The Court, relying heavily on the Opinion of Advocate-General Tesauro, found that the Commission had not established that the UK was in breach because it could not then be said that the UK courts would not interpret the different wording in conformity with the Directive. The Court made some pertinent comments on the substantive scope of the defence. In particular, it stated the defence was directed ”unreservedly, at the state of scientific and technical knowledge, including the most advanced level of such knowledge, at the time when the product in question was put into circulation”.^180^ The Advocate-General explained that the logic was for the producer “to bear only quantifiable risks, but not development risks which are, by their nature, unquantifiable”.^181^ Hence “the producer has to bear the foreseeable risks, against which he can protect himself by taking either preventive measures by stepping up experimentation and research investment or measures to cover himself by taking out civil liability insurance against any damage caused by defects in the product”.^182^ In a powerful indication that the defence was narrow in scope, the Advocate-General was clear that one isolated opinion would be sufficient to deprive the producer of the defence. The Court confirmed the defence was concerned with the most advanced state of knowledge^183^ and, whilst confirming the test was objective, gave some relief to producers by determining “the relevant scientific and technical knowledge must have been accessible”.^184^

The defence is intended to protect innovators against unknowable risks. It does not protect against products that have discoverable defects, but that were produced in haste to meet an emergency. To the extent that products carry extra risk due to emergency production, this is a matter for assessment under the defectiveness standard. As Advocate-General Tesauro noted, the practicability of measures is irrelevant to the defence.^185^ Failure to apply normal testing, or to undertake research, ought in principle not to be excused by the emergency. On one reading of the UK legislation, it could be arguable that some knowledge that producers might normally be expected to have might not similarly be expected of producers manufacturing in an emergency. However, the Court of Justice has clearly cut off such an interpretation by equating knowledge with the most advanced state of knowledge. Anything that would have been discovered by established scientific and technical testing procedures would have been discoverable. Rather than manipulate principles that may affect later cases in more normal times and circumstances, the emergency context should, if at all, be addressed as a *sui generis* matter. Possible options are considered in Section VI below.

It was noted above that the speed of production may lead to increased manufacturing defects. The defence has no application to such defects. The German Supreme Court so held when imposing liability on the producer of a carbonated mineral water bottle.^186^ Burton J in *A v National Blood Authority* ^187^ agreed, with the caveat that the defence might be available on the first occasion such a defect arose. It might be possible to quibble about this approach as the wording of the defence talks about discoverability of the defect, and as science and technology have not enabled sufficiently accurate quality assurance testing, there seems to be an argument the defence should apply. However, there seems to be a consensus that it does not apply to such manufacturing defects. This also follows the logic of the Advocate-General in that such manufacturing defects are foreseeable (probably inevitable) and so it is for the producer to determine what steps it takes to bear those risks. Certainly, increased manufacturing flaws resulting from haste of production would not be covered by the defence.

The defence is intended to shield some design defects from liability given the lack of knowledge of the risks that rendered the product defective. The key to understanding its scope is to determine how scientific and technical knowledge should be understood. Jane Stapleton has been very critical of how the Court of Justice formulated its understanding of knowledge.^188^ She is worried that a random guess by someone without scientific training could prove to be correct and undermine the producer’s defence. We have already seen there is a welter of speculation and misinformation about the SARS-CoV-2 virus. As medicines and vaccines begin to emerge, we can expect even more stories and lay theories to emerge. If one or more of these by chance turns out to be correct, does that remove the defence? It is unlikely. The Advocate-General referred to knowledge based on research.^189^ Random guesses and speculation are not research. He also said the producer’s conduct for the purpose of the defence “should be assessed using the yardstick of the knowledge of an expert in the sector”.^190^ Although there may be debate about exactly what that encompasses, it certainly excludes random guesses and lay speculation. Furthermore, the chance discoveries of facts must have been accessible to the producer.

On the other hand, the isolated opinion of one scientist that turns out to have been correct will constitute knowledge and remove the defence. The logic is that the risk is quantifiable and so the producer has the choice of undertaking further investigation to assess its exposure or take out insurance. However, the minority opinion must be accessible. Advocate-General Tesauro famously gave the example of a European producer not being expected to know the work of a Manchurian academic published in a local scientific journal in Chinese that does not go outside the region.^191^ It can be argued that this accessibility requirement was a creation of the court and that, in fact, with modern search engines, language should not be such a barrier to accessibility. As Burton J in *A v National Blood Authority* ^192^ noted, there are problems with the Manchurian example, for if Manchuria was renowned for a product, then research there may be highly relevant. The example is strikingly relevant for COVID-19. Given the Chinese origins of the virus and early experiences of how the virus affects humans, it is undeniable that Manchurian research will be vital to understanding this area.

An accessibility requirement raises a further issue of whether the knowledge that is kept private within a research laboratory of a pharmaceutical company amounts to knowledge for the purpose of the defence. In an *obiter* statement, Burton J in *A v National Blood Authority* ^193^ considered that an unpublished document or unpublished research not available to the general public, retained within the laboratory or research department of a particular company, would not be considered accessible. This would clearly be the case if the research was undertaken by another company. A company might seek to suggest that it also covered its own research that had not been made public as the Court makes an objective assessment of knowledge not linked to any particular producer. Such a result would be unfortunate and, we consider, unlikely. Where a producer in fact has the knowledge of the defect (or knowledge that enables the defect to be discovered), the defence is lost and the producer liable. The company may also, of course, be found to be negligent on such facts, but it might be able to argue that it acted with due care so long as those results it obtained remained isolated instances.

The most difficult question is to know whether the producer loses the defence at a stage before a specific risk was identified, but at a time when the means to make that discovery were known. Only rarely are discoveries accidents. The most famous example of an accidental discovery is Sir Alexander Fleming’s discovery of the antibiotic qualities of penicillin by noticing that, on returning from holiday, the mould on the culture prevented the growth of staphylococci. For the most part, though, the knowledge required for almost all new discoveries normally exists in some form. Through a process of bringing that knowledge together to test hypotheses, new knowledge is generated. One approach is to maintain that until the creative leap is made of putting the information together and testing the hypotheses, the defence should not be lost.^194^ However, the Advocate-General in *Commission v United Kingdom* talks about the defence being available only where it was impossible to discover the defect.^195^ Pugh and Pilgerstorfer therefore have argued that so long as there was the possibility of discovering the defect, the defences should not be available.^196^ The difficulty is to work out what is meant by possibility. Certainly, if there was a test or procedure available and it was simply not applied to a new vaccine or drug, it is easy to see how it can be said that there was knowledge to enable the defect to be discovered. Equally, bringing random pieces of knowledge not previously thought to be connected together with the benefit of hindsight might not be legitimately described as the basis for a possible discovery. The more challenging case is where there is relevant related information, such as about the vaccine or medicine and its effects on humans when used with viruses that are similar to COVID-19. The exact effect of the drugs will only be established after testing, but it is arguable that the knowledge to make it possible to discover the defect already existed, even if no one had thought to make the connection. The defect might not be reasonably discoverable, but it may still have been possible to discover it. It may be telling that the burden of proof is on the producer to show it was impossible to discover the defect.

## V. Regulatory interface and damages liability

Given that most consumer products are subject to some standards and that many products are in fact highly regulated, the impact of those diverse rules on the preconditions of liability has been a common feature of the case law and the relevant commentary. The relationship between regulation and liability has, however, been a complex one, even in normal times.^197^

The focus of legal analysis has traditionally been upon the impact of regulation on the notion of “defect” under the PLD. Under English law, the relevance of a product’s compliance with regulations has been examined in two recent cases. In *Wilkes*, the judge indicated that whilst regulatory approval *per se* does not constitute an automatic defence, it could nonetheless be relevant to the notion of defect, as “such approval may be evidence (and, in an appropriate case, powerful evidence) that the level of safety of the product was that which persons generally were entitled to expect”.^198^ In *Gee*, Andrews J generally agreed with the so-called “holistic” approach in *Wilkes*,^199^ warning, however, that “[t]he weight to be placed on such compliance is a matter of fact and degree in the individual case, and it may be of no relevance at all”.^200^ The judge nonetheless recognised that there may be cases where standards are highly relevant to the defect alleged (as had been indicated in *Wilkes*): “in an appropriate case compliance with … standards will have considerable weight, because they have been set at a level which the appropriate regulatory authority has determined is appropriate for safety purposes”.^201^

From these excerpts, whilst the regulatory context is of relevance,^202^ it is also clear that there is no general regulatory compliance defence in English law.^203^ A similar approach has been taken in other countries. In Germany, the Bundesgerichtshof (BGH) has adopted the position that compliance with safety regulations or standards may be important, but not a complete defence.^204^ In France, supplementary language was added to the implemented provisions so as to emphasise this point. Article 1245-9 of the French Civil Code thus provides that: “The producer may be liable for a defect even though the product has been manufactured in accordance with the rules of the trade or of existing norms or where it was the subject of an administrative authorization”. The PLD does, of course, envisage a limited defence of compliance with standards in Article 7(d), where a defect is due to the “compliance of the product with mandatory regulations”. That provision is a long way from being a broad compliance defence, as it applies only where the product defect is specifically “attributable” to compliance with the statutory requirements^205^. The defence has, as a consequence, rarely been invoked.^206^ It is true that, in the current circumstances, governmental bodies are becoming more closely involved in the production process of certain critical products than they would in normal circumstances.^207^ It would, however, be necessary for very precise standards to be prescribed by the public sector for, say, ventilators or for protective equipment, and for those very standards to be the cause of the defect, for the latter to be “*attributable to* compliance with” the regulatory requirement, as required by the statutory wording under the CPA.^208^

More radical shields from liability have also been called for. Quasi-immunities for emergency products, such as those that have been put into effect in the USA by virtue of the PREP Act (which we review below), are unlikely to be acceptable or desirable in Europe.^209^ The issue that does arise in a European context is the extent to which emergency regulatory responses such as fast-track or modified procedures, or potentially regulatory exemptions, should be taken into account in any subsequent damages claim.

From a common law position, it is well established within the tort of negligence that the fact that defendants were responding to an emergency at the time of their alleged negligence can affect the requisite standard of care.^210^ This is illustrated by cases concerning the emergency services. In *Robinson *v* Chief Constable of West Yorkshire*, a claim against the police when a passer-by was injured during the attempted arrest of a suspected drug-dealer on a busy high street, Lord Reed underlined “the importance of not imposing unrealistically demanding standards of care on police officers acting in the course of their operational duties”.^211^ Other cases have illustrated that the courts will take account of the fact that defendants were undertaking activities in the public benefit when they caused an accident.^212^ The UK legislator has also intervened to ensure that courts take into account the acts “for the benefit of society” and the dangers of requiring excessive precautions, though it is unclear whether this has added anything to the approach already adopted under the common law.^213^

From the perspective of the PLD, we have already seen above that the regulatory context is likely to be relevant to the issue of defect. To what extent, however, might that analysis be modulated or heightened in an emergency situation, where somewhat different regulatory approaches are taken. One argument might be that where regulatory demands are reduced and the processes accelerated, then the entitled expectations of safety might also be lowered. That might be seen as analogous to the common law position of modulating the standard of care in response to emergency conditions. However, it is to be noted that, in the English cases, where the defendant is not acting in an absolute emergency and does have time for considered thought, the courts have given a nuanced response as to whether it is acceptable to subject others to greater risk of injury than is normally regarded as acceptable to avert a greater harm.^214^ Moreover, the underlying theme of the regulatory response to medicines in the current climate as outlined above is that the benchmark standard of “appropriate benefit risk” is still applicable even within the context of accelerated assessment procedures. From this standpoint, should the expectations of safety not also be maintained? It might be thought that the integrity of, and confidence in, the regulatory framework for medicines is as important (if not more important) during emergencies than in normal times. That will of course need to be informed by the delicate decision of how the “fair apportionment of the risks inherent in modern technological production”^215^ should be made, and one in which the fault of the producer is not the determining factor.

## VI. Alternatives

One approach would be for the government to bear the costs of damages from products supplied in an emergency. This was the approach of the USA under the National Swine Flu Immunization Program of 1976 when plaintiffs were required to assert claims directly against the Federal government; but the exposure to limitless damages caused the USA to adopt a different approach in the National Childhood Vaccine Injury Act of 1986 that introduced a no-fault regime with caps on damages.^216^ Unless Governments steps in to become the producers of products related to COVID-19,^217^ the question arises as to whether the current emergency warrants any adaptations being made to the existing liability rules. These might take the form of the Government undertaking to indemnify producers, either contractually or based on a special law. Another option is to exempt certain products from traditional product liability laws. Finally, alternative arrangements for dealing with the claims of victims related to the emergency might be foreseen.

### 1. Indemnity

Previous health crises show that contractual indemnities have been accorded by Governments to manufacturers in order to offset risks arising from emergency products, and examples are emerging within the current pandemic.^218^ This was the case during the H1N1 pandemic in 2009. In that context, producers of vaccines sought – and obtained – very extensive indemnity clauses in respect of any injury sustained by those vaccinated due to side effects, even though such clauses might have been thought questionable under normal contract law doctrines.^219^ This approach attracted criticism from many quarters and gave rise to a critical Council of Europe report,^220^ accompanied by a strongly worded Resolution of the Parliamentary Assembly of the Council of Europe that stated *inter alia* that Member States should “ensure that the private sector does not gain undue profit from public health scares and that it is not allowed to absolve itself of liabilities with a view to privatising profits whilst sharing the risks”.^221^ As a consequence, Member States were urged, along with other measures, to “develop and implement clear national guidelines for dealing with the private sector and to co-operate with one another in negotiations with international corporations whenever necessary”. It is unclear whether this advice was heeded. More generally, the Resolution was very critical of how the pandemic had been handled in general terms and stated – somewhat ominously – that the Assembly feared that the “lack of transparency and accountability will result in a drop in confidence in the advice given by major public health institutions. This may prove disastrous in the case of a next disease of pandemic scope, which may turn out to be much more severe than the H1N1 influenza”.^222^

This salutary experience illustrates that extreme caution needs to be exercised in terms of any indemnities offered to producers and that it is preferable for States to cooperate internationally in order to obtain a better result from contractual negotiations with product manufacturers in times of crisis.

### 2. Exemption

The USA is one country that has provision for exempting producers from liability in public health emergencies. The powers are contained in the PREP Act.^223^ These have already been invoked in the current crisis. The Department of Health and Human Services issued a statutory declaration, effective from 4 February 2020, that countermeasures to which the exemption applied were “antiviral, any other drug, any biologic, any diagnostic, any other device, or any vaccine, used to treat, diagnose, cure, prevent, or mitigate COVID-19, or the transmission of SARS-CoV-2 or a virus mutating therefrom, or any device used in the administration of any such product, and all components and constituent materials of any such product”.^224^ Drugs, biologics and devices must, however, have an Emergency Use Authorisation (EUA). Pressure to add respirators led to a fourth category of countermeasures being added in the Families First Coronavirus Responses Act 2020 to cover certain respirators approved by the National Institute for Occupational Safety and Health.^225^

Those injured can make a claim under the Countermeasures Injury Compensation Program (CICP). This only covers serious physical injury, viz. one that warranted hospitalisation (though there is no requirement for actual hospitalisation) or that led to a significant loss of function or disability. However, compensation is limited to reasonable and necessary medical benefits and/or lost wages. Death benefits are also available. In the US context, the coverage of healthcare costs is significant, but this scheme is certainly less generous than the amounts attainable under a private law action. There seems to be a surprising degree of acceptance in the USA that private entities acting in the public good should benefit from these immunities.^226^ In these scenarios, it should be remembered that costs do not disappear if the harm is not compensated for; it is just left to be borne by the unfortunate victim. Such a wide-ranging carve-out of liability, leaving victims forced to accept much lower levels of state compensation, is unlikely to be acceptable in Europe and would require a significant amendment to the PLD. Europe is more likely to nudge victims towards alternative forms of compensation than to deprive victims of their private law rights.

### 3. Alternative (no-fault) compensation schemes

A more acceptable approach, from a European perspective, might be to develop (no-fault) compensation schemes. These would seek to divert claimants from traditional private law ligation to a scheme that offers easier access to compensation, albeit sometimes with lower amounts being available. Such schemes are common in relation to vaccine damage claims. Even where the victim receives lower compensation this solution may be palatable, as that outcome will be chosen by the victim who, in theory, still has the option of litigating under normal rules and conditions. Such schemes must therefore be sufficiently attractive to victims.^227^

There are several motivations for treating victims favourably in some situations so as to justify the development of a no-fault response for them.^228^ These might apply to the victims of experimental testing kits, medicines and vaccines used in an emergency if they cause harm. There might, for instance, be sympathy for the victims, where they are seen as guinea pigs for products that offered hope for the wider population. There will also be a desire not to have a potentially large number of complex cases jamming the courts with complicated and drawn-out litigation. This motive does not only focus on the plight of the victims, but there will also be a desire to reduce transaction costs by avoiding unnecessary legal and experts’ fees that often make the legal system seem expensive and inefficient. However, a major motivation of vaccine damage schemes has been the social goal of promoting vaccination. This is particularly important as vaccine programmes require high take-up rates to make immunity effective. Vaccine compensation payments help secure compliance with vaccination programmes. This is evident in Germany, where the Supreme Court first provided assistance to victims of vaccines by extending a doctrine according to which the State must compensate a citizen forced to sacrifice his/her rights for the benefit of the public.^229^ This was then codified in the *Bundesseuchengesetz* (Federal Epidemics Act; BSeuchG) of 1961.^230^ In the UK, it was noted in the *British Medical Journal* that:

The moral justification for compensation … is based on the social contract. National immunization programmes not only aim to protect the individual but also to protect society … If individuals are asked to accept a risk (even a very small one) partly for the benefit of society then it seems equitable that society should compensate the victims of occasional unlucky mishaps.^231^

Restoring public confidence in vaccination was cited by Prime Minister James Callaghan when he wrote to Lord Pearson asking that vaccine damage be considered in the Royal Commission into Personal Injury Compensation.^232^ In France, there is a special compensation scheme for injuries caused by compulsory vaccinations.^233^ Compensation is due whenever a claimant proves that an injury has been caused by a mandatory vaccine. Claims based on this special compensation scheme must be brought before a special compensation fund, ONIAM.^234^ If a claimant is not satisfied with ONIAM’s proposal of compensation, appeal can be made to an administrative court.^235^ Adopting special schemes to deal with unique product liability circumstances is a familiar technique known to many European legal systems.^236^

Acceptance of the no-fault regime may well turn on the level of benefits and ease of obtaining compensation. The French system provides full compensation in line with civil liability.^237^ The German system provides generous social security benefits. The UK has an upper limit of £120,000, and awards will often be a lot less than tort damages for similar harm. This contrasts with an average payment under the US National Childhood Vaccine Injury Act of over half a million dollars (between 2006 and 2016).^238^ It is likely that the efficiency gains from no-fault liability will include not merely lower transaction costs, but also lower payments to victims than under tort law. To make such a voluntary system a realistic alternative, the amounts need nevertheless to be sufficiently attractive. However, the conditions for recovery can even make a scheme that is less generous than the tort system appealing. Sadly, to date, the track record of state schemes has not been auspicious, with the German scheme frequently contesting causation issues,^239^ and under the UK scheme a child victim’s case was taken to the Court of Appeal by the State arguing damages should not be assessed based on future needs.^240^ Claims in the UK also often fail for want of causation: recent figures show that over 65% of claims fail for that reason.^241^ The system can also be made attractive by proving alternative dispute resolution procedures that are more accessible than the traditional courts. In the UK, there is a special tribunal, but as we have seen, there remains an appeal route to the Court of Appeal.

## VII. Conclusion

Times of crisis bring out the best and worst features of humanity. Tackling COVID-19 has demonstrated the courage of health and essential workers and the ingenuity of researchers and engineers in producing products to prevent the spread of the virus, treat the disease it causes and hopefully going forward immunise and protect the population. There have also been instances of fraudulent claims and ineffective products being rushed to market and examples of profiteering. However, for those genuinely trying to produce products for the pandemic, there is a degree of uncertainty engendered by producing at speed in the context of a virus about which knowledge is incomplete, but about which new information is emerging rapidly. There is also the prospect of the virus itself mutating. Regulatory practices have been amended to assist products entering the market as soon as reasonably possible, but the unknown for any producer is any potential downstream civil liability. Producing in an emergency will not *per se* provide a defence to civil liability, though it might be a factor to be taken into account. The above discussion has highlighted the range of issues that may be in play in any litigation making the outcome unpredictable.

Products, such as PPE, sanitisers and ventilators, have already been used or will be soon. For them, it is probably already too late to create an alternative legal regime. The risks are highest for producers of medicines and particularly vaccines. The victims of side effects from vaccines have the most pressing claims, as they were both healthy before immunisation and immunisation is needed for the greater public good. The price of any vaccine is likely to be high given the investment costs and potentially short-term need for vaccination, if the virus is supressed. If cover for legal liability has to be factored in, this will further hike the price, as insurers or self-insuring pharmaceutical companies will have to assume a worst-case scenario. Companies may press for government indemnities, but the track record has shown that these can be unfair towards states in a weak bargaining position when responding to public concerns in emergencies. An alternative is to go down the US route of exempting goods supplied in an emergency to meet pandemics, but we feel in Europe that such a loss of rights is not acceptable or justified, despite the circumstances. Instead, the option of a no-fault regime should be considered.^242^ The risks of litigation are also present for the victims, and instead of spending money on the costs of the litigation system, it may be desirable to divert victims to a compensation fund. However, the sums available need to be sufficiently high that victims are not tempted to still risk turning to litigation to top-up the award. Compensation can be based on need rather than tort principles, and economics suggests that they will be attractive if they represent a better bargain than gambling on the uncertain outcome of litigation. Some may advocate that curtailing litigation rights might be made a condition for accessing the fund, though that would need to be considered carefully and may raise human rights concerns. History shows that European governments usually respond to the needs of their citizens who suffer such tragedies.^243^ Being proactive in establishing such a fund may both make any immunisation programme effective and reduce costs by giving industry some reassurance that it is unlikely to be overwhelmed by claims. It also fits in with a philosophy that prefers to nudge citizens to make the choices the state wants rather than imposing a system and removing existing rights.

## Footnotes

1 Recognised as long ago as the writings of Confucius: L Yu, *The Analects of Confucius*, Lunyu XII. 7. (300) <http://wengu.tartarie.com/wg/wengu.php?no=300&l=Lunyu> (all web links accessed 15 May 2020).

2 For an overview, see H Li, S-M Liu, X-H Yu, S-L Tang and C-K Tang, “Coronavirus disease 2019 (COVID-19): current status and future perspectives” (2020) International Journal of Antimicrobial Agents <https://doi.org/10.1016/j.ijantimicag.2020.105951>; H Haraman et al, “Coronavirus disease 2019 (COVID-19): A literature review” (2020) Journal of Infection and Public Health <https://doi.org/10.1016/j.jiph.2020.03.019>.

3 The WHO indicates that the SARS-CoV-2 virus most probably has its ecological reservoir in bats, but jumped the species barrier via an intermediate host: <https://www.who.int/health-topics/coronavirus/who-recommendations-to-reduce-risk-of-transmission-of-emerging-pathogens-from-animals-to-humans-in-live-animal-markets>. The pangolin is a prime suspect, but no conclusive proof has yet been established: see D Cyranoski, “Mystery deepens over animal source of coronavirus” (2020) 579 Nature 18 <https://doi.org/10.1038/d41586-020-00548-w>.

4 See K Andersen et al, “The proximal origin of SARS-CoV-2” (2020) Nature Medicine <https://doi.org/10.1038/s41591-020-0820-9>.

5 <https://nextstrain.org>.

6 Team NCPERE, “Vital surveillances: the epidemiological characteristics of an outbreak of 2019 novel coronavirus diseases (COVID-19) – China” (2020) 2(8) China CDC Weekly 113.

7 WHO Scientific Brief, “Modes of transmission of virus causing COVID-19: implications for IPC precaution recommendations” (29 March 2020) < https://www.who.int/publications-detail/modes-of-transmission-of-virus-causing-covid-19-implications-for-ipc-precaution-recommendations>.

8 ibid. Research is also looking at possible transmission via animals: T Sit et al, “Infection of dogs with SARS-CoV-2” (2020) Nature <https://doi.org/10.1038/s41586-020-2334-5>.

9 In the modelling described at note 20 below, this was assumed to be 5.1 days based on N Linton et al, “Epidemiological characteristics of novel coronavirus infection: A statistical analysis of publicly available case data” (2020) medRxiv <https://doi.org/10.1101/2020.01.26.20018754>; Q Li et al, “Early Transmission Dynamics in Wuhan, China, of Novel Coronavirus-Infected Pneumonia” (2020) 382 New England Journal of Medicine 1199 <https://doi.org/10.1056/NEJMoa2001316>.

10 <https://www.cdc.gov/coronavirus/2019-ncov/symptoms-testing/symptoms.html>.

11 <https://www.who.int/news-room/q-a-detail/q-a-coronaviruses>.

12 See D Wang et al, “Hospitalized Patients with 2019 Novel Coronavirus-Infected Pneumonia in Wuhan, China” (2020) 323(11) JAMA 1061 <https://doi.org/10.1001/jama.2020.1585>.

13 M Day, “Covid-19: four fifths of cases are asymptomatic, China figures indicate” (2020) 369 BMJ m1375 <https://doi.org/10.1136/bmj.m1375>.

14 WHO Interim Guidance, “Clinical management of severe acute respiratory infection (SARI) when COVID-19 disease is suspected” (13 March 2020) <https://www.who.int/publications-detail/clinical-management-of-severe-acute-respiratory-infection-when-novel-coronavirus-(ncov)-infection-is-suspected>. The science here is emerging, see eg L Gattinoni, S Coppola, M Cressoni, M Busana, S Rossi and D Chiumello, “Covid-19 Does Not Lead to a “Typical” Acute Respiratory Distress Syndrome” (2020) 201(10) American Journal of Respiratory and Critical Care Medicine 1299 <https://doi.org/10.1164/rccm.202003-0817LE>.

15 See eg T Chen et al, “Clinical characteristics of 113 deceased patients with coronavirus disease 2019: retrospective study” (2020) 368 BMJ m1091 <https://doi.org/10.1136/bmj.m1091> (published 26 March 2020); see also “The gendered dimensions of COVID-19” (2020) 395 The Lancet 1168 <https://doi.org/10.1016/S0140-6736(20)30823-0>; ONS, “Deaths involving COVID-19, England and Wales: deaths occurring in March 2020” <https://www.ons.gov.uk/peoplepopulationandcommunity/birthsdeathsandmarriages/deaths/bulletins/deathsinvolvingcovid19englandandwales/deathsoccurringinmarch2020>.

16 See also wider studies on the role of genetics in the course of the disease; eg <https://www.ed.ac.uk/roslin/news-events/latest-news/genetics-of-covid-19-patients-is-focus-of-study>.

17 See <https://www.who.int/dg/speeches/detail/who-director-general-s-opening-remarks-at-the-media-briefing-on-covid-19---3-march-2020> and further C Wang, P Horby, F Hayden and G Gao “A novel coronavirus outbreak of global health concern” (2020) 395 The Lancet 470 <https://doi.org/10.1016/S0140-6736(20)30185-9> (assessing overall case fatality as closer to 3%).

18 <http://www.euro.who.int/en/health-topics/health-emergencies/coronavirus-covid-19/news/news/2020/3/who-announces-covid-19-outbreak-a-pandemic>.

19 See <https://www.worldometers.info/coronavirus>. As of 15 May 2020, the global number infected was approaching 4.6 million people, and deaths were over 303,000. When comparing countries, see D Spiegelhalter, “Coronavirus deaths: how does Britain compare with other countries?” *The Guardian*, 30 April 2020 <https://www.theguardian.com/commentisfree/2020/apr/30/coronavirus-deaths-how-does-britain-compare-with-other-countries>.

20 N Ferguson et al (the Imperial College COVID-19 Response Team), “Impact of non-pharmaceutical interventions (NPIs) to reduce COVID19 mortality and healthcare demand” (16 March 2020) <https://doi.org/10.25561/77482>.

21 ibid, 4. Higher estimates have been used in later work: eg 2.4–3.3 in note 22 below; and 3.87 (3.01–4.66) in note 23 below. See further Y Liu, A Gayle, A Wilder-Smith and J Rocklöv, “The reproductive number of COVID-19 is higher compared to SARS coronavirus” (2020) 27(2) Journal of Travel Medicine taaa021 <https://doi.org/10.1093/jtm/taaa021>; E Mahase, “Covid-19: What is the R number?” (2020) 369 BMJ m1891 <https://www.bmj.com/content/bmj/369/bmj.m1891.full.pdf> (reporting that estimates vary between 0.4 and 4.6).

22 P Walker et al (the Imperial College COVID-19 Response Team), “The Global Impact of COVID-19 and Strategies for Mitigation and Suppression” (26 March 2020) <https://doi.org/10.25561/77735> estimated that, in the absence of interventions, COVID-19 would have resulted in 7 billion infections and 40 million deaths globally in 2020. See also N Jewell, J Lewnard and B Jewell, “Predictive Mathematical Models of the COVID-19 Pandemic” (2020) JAMA <https://doi.org/10.1001/jama.2020.6585>.

23 S Flaxman et al (the Imperial College COVID-19 Response Team), “Estimating the number of infections and the impact of non-pharmaceutical interventions on COVID-19 in 11 European countries” (30 March 2020) <https://doi.org/10.25561/77731>. See also the IHME COVID-19 projections <https://covid19.healthdata.org/united-kingdom>; C Jarvis et al, “Impact of physical distance measures on transmission in the UK” (31 March 2020, forthcoming) available at <https://cmmid.github.io/topics/covid19/reports/LSHTM-CMMID-20200401-CoMix-social-contacts.pdf>; Mahase, supra, note 21.

24 Ferguson et al, supra, note 20.

25 D Adam, “Special Report: The simulations driving the world’s response to COVID-19” (2020) Nature <https://doi.org/10.1038/d41586-020-01003-6>. For further information on contact tracing, see C Baraniuk, “Covid-19 contract tracing: a briefing” (2020) 369 BMJ m1859 <https://www.bmj.com/content/bmj/369/bmj.m1859.full.pdf>.

26 See eg A. Kucharski et al, CMMID COVID-19 Working Group, “Effectiveness of isolation, testing, contact tracing and physical distancing on reducing transmission of SARS-CoV-2 in different settings” (2020) medRxiv <https://doi.org/10.1101/2020.04.23.20077024>.

27 (2005) 42 USC §247d-6d. This is discussed in more detail in Section VI below.

28 MHRA: <https://www.gov.uk/guidance/mhra-regulatory-flexibilities-resulting-from-coronavirus-covid-19>; EMA: <https://www.ema.europa.eu/en/news/covid-19-how-ema-fast-tracks-development-support-approval-medicines-vaccines>.

29 Directive 2001/83/EC.

30 See <https://www.ema.europa.eu/en/news/covid-19-developers-medicines-vaccines-benefit-free-scientific-advice>.

31 <https://www.ema.europa.eu/en/human-regulatory/research-development/prime-priority-medicines>.

32 <https://www.ema.europa.eu/en/documents/other/call-pool-eu-research-resources-large-scale-multi-centre-multi-arm-clinical-trials-against-covid-19_en.pdf>.

33 <https://www.ema.europa.eu/en/implications-coronavirus-disease-covid-19-methodological-aspects-ongoing-clinical-trials>.

34 <https://www.gov.uk/guidance/mhra-regulatory-flexibilities-resulting-from-coronavirus-covid-19>.

35 ibid.

36 The Medical Devices Regulations 2002 (SI 2002 No 618, as amended)

37 <https://www.gov.uk/topic/medicines-medical-devices-blood/medical-devices-regulation-safety>.

38 See eg the WHO Director General’s advice to governments to “test, test, test” (16 March 2020) <https://www.who.int/dg/speeches/detail/who-director-general-s-opening-remarks-at-the-media-briefing-on-covid-19---16-march-2020>.

39 WHO Interim Guidance, “Global surveillance for COVID-19 caused by human infection with COVID-19 virus” (20 March 2020) <https://apps.who.int/iris/handle/10665/331506>.

40 Even where testing capacity cannot meet needs, current WHO guidance provides for the prioritisation of testing of health workers: WHO Interim Guidance, “Laboratory testing strategy recommendations for COVID-19” (21 March 2020) <https://apps.who.int/iris/handle/10665/331509>.

41 WHO Interim Guidance, “Laboratory testing for coronavirus disease 2019 (COVID-2019) in suspected human cases” (2 March 2020) <https://apps.who.int/iris/handle/10665/331329>.

42 See C Sheridan, “Coronavirus and the race to distribute reliable diagnostics” (2020) Nature Biotechnology <https://doi.org/10.1038/d41587-020-00002-2>.

43 By Public Health England: <https://www.gov.uk/government/news/phe-novel-coronavirus-diagnostic-test-rolled-out-across-uk>.

44 Ten rapid antigen tests have been awarded a CE mark: see ECDC Technical Report, “An overview of the rapid test situation for COVID-19 diagnosis in the EU/EEA” (1 April 2020) <https://www.ecdc.europa.eu/sites/default/files/documents/Overview-rapid-test-situation-for-COVID-19-diagnosis-EU-EEA.pdf>.

45 <https://www.sciencemediacentre.org/expert-comments-about-reagents-needed-for-covid-19-swab-pcr-testing>.

46 <https://www.theguardian.com/world/2020/mar/27/coronavirus-test-kits-withdrawn-spain-poor-accuracy-rate>.

47 <https://www.ft.com/content/f3435779-a706-45c7-a7e2-43efbdd7777b>; <https://www.nytimes.com/2020/04/16/world/europe/coronavirus-antibody-test-uk.html>.

48 See Ministry of Commerce, “General Administration of Customs and the National Medical Product Administration (NMPA) Notice No. 5 of 2020: Notice on the Orderly Conducting of Medical Materials Export”. This notice applies to COVID-19 detection reagents, medical masks, protective clothing, ventilators and infrared thermometers <https://uk.reuters.com/article/us-health-coronavirus-china-testkits/china-clamps-down-on-coronavirus-test-kit-exports-after-accuracy-questioned-idUKKBN21J51S>.

49 Over 60 CE-marked rapid antibody tests are currently on the market: see ibid, but none are for home use: <https://www.gov.uk/guidance/guidance-on-coronavirus-covid-19-tests-and-testing-kits>.

50 “[T]here is little information on the accuracy of the tests, or on how a patient’s antibody response develops or changes during COVID-19 infection. It is not known whether either a positive or negative result is reliable”: <https://www.gov.uk/government/publications/covid-19-rapid-tests-for-use-in-community-pharmacies-or-at-home/covid-19-rapid-tests-for-use-in-community-pharmacies-or-at-home>. At the time of writing, Public Health England had just approved an “Elecsys Anti-SARS-CoV-2 serology test” (processed in laboratories) produced by Roche: see <https://www.theguardian.com/world/2020/may/13/public-health-england-approves-roche-test-for-coronavirus-antibodies> and <https://www.roche.com/media/releases/med-cor-2020-04-17.htm>.

51 N Okba et al, “SARS-CoV-2 specific antibody responses in COVID-19 patients” (2020) medRxiv <https://doi.org/10.1101/2020.03.18.20038059>; see also the comments of Dr Maria van Kerkhove, WHO technical lead for COVID-19, press conference 17 April 2020, on evidence that antibody tests can show an individual is immune or protected from infection <https://www.sciencemediacentre.org/expert-reaction-to-comments-made-by-dr-maria-van-kerkhove-at-the-who-that-there-is-no-evidence-antibody-tests-can-show-that-an-individual-is-immune-or-is-protected-from-the-infection>; F Amanat et al, “A serological assay to detect SARS-CoV-2 seroconversion in humans” (2020) medRxiv <https://doi.org/10.1101/2020.03.17.20037713>.

52 See in general <https://www.gov.uk/government/publications/in-vitro-diagnostic-medical-devices-guidance-on-legislation>.

53 <https://www.gov.uk/guidance/guidance-on-coronavirus-covid-19-tests-and-testing-kits>; cf the FDA approach: “the FDA does not intend to object to the distribution and use of serology tests to identify antibodies to SARS-CoV-2 where the test has been validated, notification is provided to the FDA, and warning statements are included with the tests, for example, noting the test has not been reviewed by the FDA and results from antibody testing should not be used as the sole basis to diagnose or exclude SARS-CoV-2 infection or to inform infection status” <https://www.fda.gov/news-events/press-announcements/coronavirus-covid-19-update-fda-provides-more-regulatory-relief-during-outbreak-continues-help>.

54 See the six-month ban in Belgium: <https://www.afmps.be/fr/news/coronavirus_interdiction_de_vente_de_tests_rapides_de_diagnostic_vu_le_risque_de_mauvaise>.

55 ECDC Technical Report, supra, note 44.

56 Eg the obligation from 6 April 2020 to wear face masks in Austrian supermarkets and chemists as prescribed: <https://www.bundeskanzleramt.gv.at/bundeskanzleramt/nachrichten-der-bundesregierung/2020/bundeskanzler-sebastian-kurz-mund-nasen-schutz-wird-pflicht-abstand-halten-weiterhin-wichtig.html; https://orf.at/corona/stories/3160118>.

57 See the answer of Dr Michael Ryan at the WHO press conference of 30 March 2020 <https://www.who.int/docs/default-source/coronaviruse/transcripts/who-audio-emergencies-coronavirus-press-conference-full-30mar2020.pdf?sfvrsn=6b68bc4a_2>, and more generally <https://www.who.int/emergencies/diseases/novel-coronavirus-2019/advice-for-public/when-and-how-to-use-masks>.

58 N. Leung et al, “Respiratory virus shedding in exhaled breath and efficacy of face masks” (2020) Nature Medicine <https://doi.org/10.1038/s41591-020-0843-2>; Z Guo et al, “Aerosol and surface distribution of severe acute respiratory syndrome coronavirus 2 in hospital wards, Wuhan, China, 2020” (2020) Emerging Infectious Diseases <https://doi.org/10.3201/eid2607.200885>; <https://www.aalto.fi/en/news/researchers-modelling-the-spread-of-the-coronavirus-emphasise-the-importance-of-avoiding-busy>.

59 T Greenhalgh, M Schmid, T Czypionka, D Bassler and L Gruer, “Face masks for the public during the COVID-19 crisis” (2020) 369 BMJ m1435 <https://doi.org/10.1136/bmj.m1435>.

60 WHO Interim Guidance, “Rational use of personal protective equipment (PPE) for coronavirus disease (COVID-19)” (19 March 2020) <https://apps.who.int/iris/handle/10665/331498>.

61 <https://www.gov.uk/government/publications/wuhan-novel-coronavirus-infection-prevention-and-control/covid-19-personal-protective-equipment-ppe>.

62 ibid. See also A Rimmer, “Covid-19: Third of surgeons do not have adequate PPE, royal college warns” (2020) 369 BMJ m1492 <https://doi.org/10.1136/bmj.m1492>; D Kamerow, “Covid-19: the crisis of personal protective equipment in the US” (2020) 369 BMJ m1367 <https://doi.org/10.1136/bmj.m1367>. 3M announced it had doubled its global output of N95 respirators to over 1.1 billion per year, and would seek to increase production to 2 billion within 12 months: <https://news.3m.com/blog/3m-stories/3m-responds-2019-novel-coronavirus>.

63 <https://www.washingtonpost.com/business/2020/04/03/white-house-scrambles-scoop-up-medical-supplies-angering-canada-germany>.

64 See eg relaxation of import duty and VAT rules: <https://www.gov.uk/guidance/pay-no-import-duty-and-vat-on-medical-supplies-equipment-and-protective-garments-covid-19>; <https://ec.europa.eu/taxation_customs/covid-19-taxud-response/covid-19-waiving-vat-and-customs-duties-vital-medical-equipment_en>.

65 <https://ec.europa.eu/docsroom/documents/40521; see also <https://ec.europa.eu/commission/presscorner/detail/en/ip_20_558>.

66 <https://www.geekwire.com/2020/maker-mask-launches-seattle-using-3d-printing-technology-produce-protective-gear>; <https://www.bbc.co.uk/news/technology-52111522>.

67 <https://ec.europa.eu/docsroom/documents/40562>.

68 See in particular G Kampf, D Todt, S Pfaender and E Steinmann, “Persistence of coronaviruses on inanimate surfaces and their inactivation with biocidal agents” (2020) 104 Journal of Hospital Infection 246 <https://doi.org/10.1016/j.jhin.2020.01.022> (from which the data cited here are taken).

69 N Van Doremalen et al, “Aerosol and Surface Stability of SARS-CoV-2 as Compared with SARS-CoV-1” (2020) New England Journal of Medicine <https://doi.org/10.1056/NEJMc2004973>: “SARS-CoV-2 was more stable on plastic and stainless steel than on copper and cardboard, and viable virus was detected up to 72 hours after application to these surfaces”.

70 WHO Guidance, “Infection prevention and control during health care when novel coronavirus (nCoV) infection is suspected” (19 March 2020), <https://www.who.int/publications-detail/infection-prevention-and-control-during-health-care-when-novel-coronavirus-(ncov)-infection-is-suspected-20200125>; see also WHO Technical Brief, “Water, sanitation, hygiene and waste management for the COVID-19 virus” (3 March 2020) < https://www.who.int/publications-detail/water-sanitation-hygiene-and-waste-management-for-the-covid-19-virus-interim-guidance>.

71 70% ethanol also being recommended by the WHO for disinfecting small surfaces: WHO. Annex G. *Use of Disinfectants: Alcohol and Bleach. Infection Prevention and Control of Epidemic- and Pandemic-Prone Acute Respiratory Infections in Health Care* (Geneva, WHO Press 2014) pp 65–66.

72 A Siddharta et al, “Virucidal Activity of World Health Organization-Recommended Formulations Against Enveloped Viruses, Including Zika, Ebola, and Emerging Coronaviruses” (2017) 215 Journal of Infectious Diseases 902 <https://doi.org/10.1093/infdis/jix046>.

73 G Kampf, “Efficacy of ethanol against viruses in hand disinfection” (2018) 98(4) Journal of Hospital Infection 331 <https://doi.org/10.1016/j.jhin.2017.08.025>.

74 <https://www.theguardian.com/business/2020/mar/18/brewdog-begins-making-hand-sanitiser-shortages-uk>; see also <https://news.trust.org/item/20200326161350-adoqk>.

75 <https://ec.europa.eu/docsroom/documents/40523>.

76 <https://pipelawsredesign.lexblogplatform.com/wp-content/uploads/sites/451/2020/03/FDA-Hand-Sanitizer-Guidance.pdf>.

77 Supra, note 14.

78 Many countries are building temporary hospital space in response. See eg <https://www.england.nhs.uk/2020/04/nhs-to-build-more-nightingale-hospitals-as-london-set-for-opening>.

79 There have also been reports of incidents with ventilators in Russia catching fire: see BBC News (12/3 May 2020) <https://www.bbc.co.uk/news/world-europe-52629781; https://www.bbc.co.uk/news/world-us-canada-52649018>.

80 <https://www.theguardian.com/society/2020/apr/02/london-hospital-almost-runs-out-oxygen-coronavirus-patients>.

81 See eg Drägerwerk’s record number of orders: <https://www.spiegel.de/international/germany/german-ventilator-manufacturer-absolutely-mission-impossible-a-549d1e18-8c21-45f1-846f-cf5ca254b008>.

82 See <https://www.france24.com/en/20200331-france-s-macron-visits-coronavirus-mask-factory-as-health-workers-complain-of-acute-shortage>.

83 Consisting of Airbus, BAE Systems, Ford Motor Company, GKN Aerospace, High Value Manufacturing Catapult, Inspiration Healthcare Group, Meggitt, Penlon, Renishaw, Rolls-Royce, Siemens Healthineers and Siemens UK, Smiths Group, Thales, Ultra Electronics, Unilever, UK-based F1 teams: Haas F1, McLaren, Mercedes, Red Bull Racing, Racing Point, Renault Sport Racing, Williams, Accenture, Arrow Electronics, Dell Technologies, Microsoft and PTC. See <https://www.gov.uk/government/news/first-new-ventilators-to-roll-off-production-line-this-weekend-as-industry-answers-call-to-step-up-manufacturing>.

84 See <https://www.forbes.com/sites/daviddawkins/2020/03/26/billionaire-james-dyson-confirms-initial-order-of-10000-ventilators-will-made-in-britain-at-raf-hullavington/#4d77be4a1660> and further <https://www.theguardian.com/technology/2020/mar/26/from-vacuum-cleaners-to-ventilators-can-dyson-make-the-leap>.

85 <https://www.theguardian.com/world/2020/mar/30/f1-team-helps-build-new-uk-breathing-aid-for-covid-19-patients>.

86 <https://www.afmps.be/fr/news/coronavirus_lignes_directrices_pour_limpression_3d_daccessoires_dappareils_respiratoires>.

87 EU Commission, Proposal for a Regulation of the European Parliament and of the Council amending Regulation (EU) 2017/745 on medical devices as regards the dates of application of certain of its provisions COM(2020) 144 final.

88 <https://ec.europa.eu/docsroom/documents/40607?locale=en>.

89 See further Art 11(13) Directive 93/42/EEC concerning medical devices.

90 See Regulations 12(5), 26(3) and 39(2). See generally <https://www.gov.uk/guidance/exemptions-from-devices-regulations-during-the-coronavirus-covid-19-outbreak?>. However, it would seem on the latest information that non-CE-marked devices have not had to be used, at least in the first wave of the crisis in the UK.

91 <https://www.gov.uk/government/publications/coronavirus-covid-19-ventilator-supply-specification/rapidly-manufactured-ventilator-system-specification>.

92 See eg Z Jin et al, “Structure of M^pro^ from COVID-19 virus and discovery of its inhibitors” (2020) Nature <https://doi.org/10.1038/s41586-020-2223-y>.

93 WHO Scientific Brief, “Off-label use of medicines for COVID-19” (31 March 2020) <https://apps.who.int/iris/handle/10665/331640>.

94 A trial by the US National Institute of Allergy and Infectious Diseases (NIAID) produced preliminary results indicating patients receiving remdesivir had a 31% faster time to recovery compared with placebo: <https://www.niaid.nih.gov/news-events/nih-clinical-trial-shows-remdesivir-accelerates-recovery-advanced-covid-19> (29 April 2020). But contrast Y Wang et al, “Remdesivir in adults with severe COVID-19: a randomised, double-blind, placebo-controlled, multicentre trial” (2020) The Lancet <https://doi.org/10.1016/S0140-6736(20)31022-9>.

95 <https://www.who.int/emergencies/diseases/novel-coronavirus-2019/global-research-on-novel-coronavirus-2019-ncov/solidarity-clinical-trial-for-covid-19-treatments>.

96 L Caly, J Druce, M Catton, D Jans and K Wagstaff, “The FDA-approved drug Ivermectin inhibits the replication of SARS-CoV-2 *in vitro*” (2020) Antiviral Research <https://doi.org/10.1016/j.antiviral.2020.104787>.

97 <https://www.nice.org.uk/covid-19>.

98 <https://www.gov.uk/guidance/clinical-trials-applications-for-coronavirus-covid-19>.

99 <https://www.gov.uk/guidance/managing-clinical-trials-during-coronavirus-covid-19>.

100 <https://www.fda.gov/media/136238/download>.

101 Tweet, 21 March 2020, 2.13 pm: “HYDROXYCHLOROQUINE & AZITHROMYCIN, taken together, have a real chance to be one of the biggest game changers in the history of medicine. The FDA has moved mountains – Thank You! Hopefully they will BOTH (H works better with A, International Journal of Antimicrobial Agents)…….be put in use IMMEDIATELY. PEOPLE ARE DYING, MOVE FAST…”. This was based on a small non-randomised trial in France (P Gautret et al, “Hydroxychloroquine and azithromycin as a treatment of COVID-19: results of an open-label non-randomized clinical trial” (2020) International Journal of Antimicrobial Agents <https://doi.org/10.1016/j.ijantimicag.2020.105949>). See also White House Briefing 4 April 2020: “What do you have to lose? Take it … I really think they should take it. But it’s their choice. And it’s their doctor’s choice or the doctors in the hospital. But hydroxychloroquine. Try it, if you’d like” <https://www.nytimes.com/2020/04/04/health/coronavirus-drug-trump-hydroxycholoroquine.html>; cf the views of Dr Anthony Fauci, Director of the National Institute of Allergy and Infectious Diseases, at the same briefing. See further the FDA Update of 19 March 2020 <https://www.fda.gov/news-events/press-announcements/coronavirus-covid-19-update-fda-continues-facilitate-development-treatments?utm_campaign=031920_PR_Coronavirus%20%28COVID-19%29%20Update%3A%20Chloroquine&utm_medium=email&utm_source=Eloqua>.

102 C Piller, “‘This is insane!’ Many scientists lament Trump’s embrace of risky malaria drugs for coronavirus” (2020) Science <https://www.sciencemag.org/news/2020/03/insane-many-scientists-lament-trump-s-embrace-risky-malaria-drugs-coronavirus#>. See also the consequent challenges caused for patients with arthritis and systemic lupus erythematosus: B Owens, “Excitement around hydroxychloroquine for treating COVID-19 causes challenges for rheumatology” (2020) The Lancet Rheumatology <https://doi.org/10.1016/S2665-9913(20)30089-8>.

103 See R Ferner and J Aronson, “Chloroquine and hydroxychloroquine in Covid-19” (2020) 369 BMJ <https://doi.org/10.1136/bmj.m1432>, noting that “no drug is guaranteed to be safe, and wide use of hydroxychloroquine will expose some patients to rare but potentially fatal harms, including serious cutaneous adverse reactions, fulminant hepatic failure, and ventricular arrhythmias (especially when prescribed with azithromycin); overdose is hazardous and difficult to treat.” See also S Jaffe, “Regulators split on antimalarials for COVID-19” (2020) 395 The Lancet 1179 <https://doi.org/10.1016/S0140-6736(20)30817-5>.

104 See WHO Scientific Brief, supra, note 93 and WHO, “Guidance for Managing Ethical Issues in Infectious Disease Outbreaks” (2016) <https://www.who.int/ethics/publications/infectious-disease-outbreaks/en>.

105 “Coronavirus drugs trials must get bigger and more collaborative” (2020) 581 Nature 120 <https://doi.org/10.1038/d41586-020-01391-9>.

106 See eg Q Gao et al, “Rapid development of an inactivated vaccine for SARS-CoV-2” (2020) bioRxiv <https://doi.org/10.1101/2020.04.17.046375>.

107 <https://www.who.int/blueprint/priority-diseases/key-action/Novel-Coronavirus_Landscape_nCoV-4april2020.pdf?ua=1>.

108 Eg GSK and Sanofi: see <https://www.sanofi.com/en/media-room/press-releases/2020/2020-04-14-13-00-00>.

109 See A Thomson, R Sylvester, C Smyth and O Wright, “Coronavirus vaccine could be ready for September”, *The Times*, 11 April 2020 <https://www.thetimes.co.uk/article/coronavirus-vaccine-could-be-ready-by-september-flmwl257x>.

110 See P Newton and K Bond, “COVID-19 and risks to the supply and quality of tests, drugs and vaccines” (2020) The Lancet <https://doi.org/10.1016/S2214-109X(20)30136-4>.

111 K Duan et al, “The feasibility of convalescent plasma therapy in severe COVID-19 patients: a pilot study” (2020) medRxiv <https://doi.org/10.1101/2020.03.16.20036145>; C Shen et al, “Treatment of 5 Critically Ill Patients With COVID-19 With Convalescent Plasma” (2020) JAMA <https://doi.org/10.1001/jama.2020.4783>.

112 For the avoidance of any doubt, nothing in this article should be understood or taken to imply any safety issues, wrongdoing, likely liability or regulatory default in respect of the products being manufactured and developed by individuals, companies, consortia or other organisations named in the text.

113 <https://www.fda.gov/consumers/consumer-updates/beware-fraudulent-coronavirus-tests-vaccines-and-treatments>.

114 See Operation Pangea XIII: <https://www.gov.uk/government/news/coronavirus-global-crackdown-sees-a-rise-in-unlicenced-medical-products-related-to-covid-19>.

115 <https://www.ncsc.gov.uk/news/public-urged-to-flag-covid-19-threats-new-campaign>.

116 <https://edition.cnn.com/2020/03/02/tech/amazon-coronavirus-products/index.html>.

117 See Directive 2005/29/EC concerning unfair business-to-consumer practices in the internal market (OJ No L 149, 11.6.2005, p22). False information about the main characteristics of a product (including risks and benefits), which is likely to cause the average consumer to take a transactional decision he or she would not otherwise have done, is regarded as a misleading commercial practice under Art 6(1)(b). For further information, see the Commission Guidance SWD/2016/0163 final. In England, this Directive is implemented by the Consumer Protection from Unfair Trading Regulations 2008/1277, which, in addition to specifying the authorities that have the duty and power to enforce the Regulations (Reg 19), also provides for consumers’ rights to redress (including a claim for damages in certain circumstances); see Section II.4.A.

118 To use Burton J’s language in *A & others v National Blood Authority* [2001] 3 All ER 289 (QBD).

119 Although see Thomson et al, supra, note 109.

120 85/374/EEC OJ L 210/29 of 7 August 1985.

121 No such defence is to be found in Art 7 or elsewhere in the PLD.

122 Hansard 20 January 1987 HL (speech in the context of preferring inclusion of the optional development risks defence).

123 PLD Arts 3(1), (2); see the discussion at *Aventis Pasteur SA v OB (Case C-358/08)* [2010] 2 CMLR 16 (CJEU) AG§35.

124 PLD Art 3(3) and *OB (Case C-358/08)*, op. cit., at AG§98.

125 See Thomson et al, supra, note 109, and the discussion below concerning vaccines.

126 The pubic nature of the defendant in *Veedfald* (see note 127 below) made no difference: see §21.

127 *Veedfald v Arhus Amtskommune (Case C-203/99)* [2003] 1 CMLR 41 (CJEU).

128 PLD Art 7(c).

129 *Veedfald*, supra, note 127, §21 (cf AG§26). Nor did the defence in PLD Art 7(a) apply: the product was put into circulation because although it did not leave the hospital’s sphere of control, the person for whom it was intended was obliged to bring himself into that sphere of control (see §§16–18).

130 In many jurisdictions, employers also face additional considerations quite apart from those as producers of a product they might manufacture. In England, an employer owes duties of care at common law and under health and safety legislation towards their employees, including in respect of equipment and PPE for use at work; see, eg, A Cowper, “What the law says about PPE responsibility” (2020) 369 BMJ m1718 <https://doi.org/10.1136/bmj.m1718>. Furthermore, even where the employer is not themselves a producer but supplies equipment produced by a third party to an employee for the purposes of the employer’s business, under the Employers’ Liability (Defective Equipment) Act 1969, the employer is liable just as much as the third-party producer, where an employee suffers injury in the course of employment due to the equipment being defective.

131 For more details, see G Howells, C Twigg-Flesner and C Willett, “Protecting the Values of Consumer Law in the Digital Economy: The Case of 3D-Printing”, in A De Franceschi, R Schulze, M Graziadei, O Pollicino, F Riente, S Sica and P Sirena (eds), *Digital Revolution – Challenges for Law: Data Protection, Artificial Intelligence, Smart Products, Blockchain Technology and Virtual Currencies* (Munich, C. H. Beck 2019) and, from a US perspective, see J Beck and M Jacobson, “3D Printing: What Could Happen to Products Liability When Users (and Everyone Else in Between) Become Manufacturers” (2017) 18 Minnesota Journal of Law, Science & Technology 143.

132 Howells et al, supra, note 131, at 16.

133 PLD Art 2; in 1999, an exclusion for primary agricultural products and game was repealed.

134 For a discussion, see S Whittaker, “European Product Liability and Intellectual Products” (1989) 105 Law Quarterly Review 125; S Whittaker, *Liability for Products* (Oxford, Oxford University Press 2005) at 477; G Howells, C Twigg-Flesner and C. Willett, “Product Liability and Digital Products”, in T Synodinou, P Jougleux, C Markou and T Prastitou (eds), *EU Internet Law – Regulation and Enforcement* (Berlin, Springer 2017); P Rott, *Rechtspolitischer Handlungsbedarf im Haftungsrecht, insbesondere für digitale Anwendungen – Gutachten im Auftrag des vzbv* (Berlin, Verbraucherzentrale Bundesverband e.V. 2018).

135 This is a topic that has been discussed by the Expert Group on Liability and New Technologies: see <https://ec.europa.eu/transparency/regexpert/index.cfm?do=groupDetail.groupDetail&groupID=3592&NewSearch=1&NewSearch=1> and Expert Group on Liability and New Technologies, “Liability for Artificial Intelligence and Other Emerging Digital Technologies” (2019) <https://doi.org/10.2838/573689>; see further Howells et al, supra, note 131. In respect of contractual remedies, note that Chapter 3 of the Consumer Rights Act 2015 and the Digital Content Directive 2019/770 extend conformity rules to software.

136 See the words “*notamment*” and “*insbesondere*” in the French- and German-language versions of the PLD; see also *A v National Blood*, supra, note 118. at §§34ii, §63.

137 *Boston Scientific Medizintechnik GmbH v AOK Sachsen-Anhalt (Case C-503/13, 504/13)* [2015] 3 CMLR 173 (CJEU) §§37–38, AG§§28–29. See to this effect the earlier English case law: *A v National Blood*, supra, note 118, §31(vii). See further now *Wilkes* and *Gee* (both cited below).

138 *Boston Scientific*, supra, note 137, §40, AG§30; see similarly *W & others v Sanofi Pasteur MSD SNC (Case C-621/15)* ECLI:EU:C:2017:484 (CJEU) §§23, 32, 41.

139 See D Fairgrieve and M Pilgerstorfer, “European Product Liability after Boston Scientific: An Assessment of the Court’s Judgment on Defect, Damage and Causation” (2017) European Business Law Review 879; see also *Busby v Berkshire Bed Co Ltd* [2018] EWHC 2976, §140: “The reference within [the] subsection is risk as opposed to hazard”.

140 See J-S Borghetti, *La Responsabilité du Fait des Produits: Etude de Droit Comparé* (Paris, LGDJ 2004) at 447, §451; Fairgrieve and Pilgerstorfer, supra, note 139, at 887, 889–90.

141 Similar issues arise in respect of other theories of liability for products. In contractual liability, for example, “safety” is an aspect of the quality of goods for the purposes of assessing satisfactory quality: see Consumer Rights Act 2015 (CRA) s9(3)(d). A similar test is applied whether the reasonable person would consider the goods satisfactory taking account of all relevant circumstances: see s9(2) CRA. As a matter of negligence in England, the utility, desirability or social benefit of the activity causing harm falls to be considered as part of the fault analysis both at common law and under s1 Compensation Act 2006 and s2 of the Social Action, Responsibility and Heroism Act 2015.

142 *A v National Blood*, supra, note 118, §§35, 57, 63, 68, 71–72.

143 See *Abouzaid v Mothercare (UK) Limited* (2000) Unreported Case No. B3/2000/2273 (CA) §43; *A v National Blood*, supra, note 118, §§35, 57, 71; and *Gee & others v DePuy International Ltd* [2018] EWHC 1208, [2018] Med. L.R. 347 (QBD) §138, §140.

144 *Wilkes v Depuy International Ltd* [2018] QB 627 (QBD), §78; see also *Gee*, supra, note 143, §143 and *AH v Greater Glasgow Health Board* [2018] SLT 535, §114.

145 See *A v National Blood*, supra, note 118, §§36, 67.

146 ibid, §§63, 68, 72; *B & others v McDonald’s Restaurants Ltd* [2002] EWHC 490 (QBD), §73(d).

147 *Wilkes*, supra, note 144, §96; *Gee*, supra, note 143, §§145–46.

148 See *Commission v United Kingdom (Case C-300/95)* [1997] 3 CMLR 923 (CJEU), AG§§16–17; *Novo Nordisk Pharma GmbH v S (Case C-310/13)* [2014] ECR 0 (CJEU), AG§18.

149 *Boston Scientific*, supra, note 137, AG§38; see also G Calabresi and JT Hirschoff, “Toward a Test for Strict Liability in Torts” (1972) 81 The Yale Law Journal 1055, 1060–61; H Koziol, “Product Liability: Conclusions from a Comparative Perspective” in H Koziol et al (eds), *Product Liability: Fundamental Questions in a Comparative Perspective* (Berlin De Gruyter 2017), 532–34.

150 *Boston Scientific*, supra, note 137, AG§§34-5, 37, 63; *Commission v United Kingdom*, supra, note 148, AG§§16–17.

151 *Boston Scientific*, supra, note 137, §41, AG§§3, 54.

152 During the preparation of the PLD, the American Chamber of Commerce suggested to the Commission that the definition of defectiveness “should be amended to include specific language concerning unavoidably unsafe but useful products, such as explosives, selective weed killers, pesticides and prescription pharmaceuticals, and that this language should address a benefit-to-risk evaluation of the product”: see further *A v National Blood*, supra, note 118, §§35(i), 43(i).

153 *A v National Blood*, supra, note 118, §§68–69, 71.

154 *Wilkes*, supra, note 144, §§13, 65–66, 82 and *Gee*, supra, note 143, §161; see also *Seroxat Group Litigation* [2019] EWCA Civ 1926 at §8.

155 See eg Deutscher Bundestag, *Entwurf eines Gesetzes über die Haftung für fehlerhafte Produkte Drucksache 11/2447* (1988), 18; *Airbag* (Urteil vom 16.6.2009 VI ZR 107/08) NJW 2009, 2952 (BGH), §17–18; *Cherry Cake* (Urteil vom 17.3.2009 VI ZR 176/08) NJW 2009. 1669 (BGH), §§10–11.

156 *W v Sanofi*, supra, note 138, AG§§85–88.

157 *Gee*, supra, note 143, §§149–50.

158 ibid, §§162–63.

159 Or else the development risks defence might apply; see further Section IV below.

160 A parallel arises when considering contractual liability: public statements about the specific characteristics of the goods made by the producer go to quality pursuant to CRA s9(5).

161 *A v National Blood*, supra, note 118, §§55–56, 65.

162 §§106–08; see also *Gee*, supra, note 143, §§168–69.

163 PLD Art 1.

164 PLD Art 4.

165 See *W v Sanofi*, supra, note 138, §§25–27; R Goldberg, *Causation and Risk in the Law of Torts: Scientific Evidence and Medicinal Product Liability* (Oxford, Hart 1999) at 23; cf S Whittaker, “The EEC Directive on Product Liability” (1985) 5 Yearbook of European Law 233: “semi-autonomous”.

166 *W v Sanofi*, supra, note 138, §§30–32, §43 and AG§§42–51 (distinguishing between judicial and scientific assessments of causation).

167 *W v Sanofi*, supra, note 138, §37.

168 *Gee*, supra, note 143, §180; cf Fairgrieve and Pilgerstorfer, supra, note 139.

169 Whilst adopted by consent in *XYZ v Schering Health Care Ltd* [2002] EWHC 1420 (QBD) §20(1), §21, it remains controversial as to whether relative risk >2 is a requirement, and if so, and if so, whether there is any discretion as to its application: see RS Carruth and BD Goldstein, “Relative Risk Greater than Two in Proof of Causation in Toxic Tort Litigation” (2001) 41 Jurimetrics 195, 199; R Jay, “Standards of Proof in Law and Science: Distinctions Without a Difference?” (2016) Journal of Personal Injury Law 1. Cases applying relative risk >2 include *Sienkiewicz v Greif (UK) Ltd* [2010] QB 370 (CA) §23, but on appeal the Supreme Court was more cautious: *Sienkiewicz v Greif (UK) Ltd* [2011] 2 AC 229 (SC) §§76, 78, 90, 121, 160–63, §§85–89. A broader assessment is to be seen in *Rich v Hull and East Yorkshire Hospitals NHS Trust* [2016] Med LR 33 (QBD) §§142–43, 170, 171, 175, 181, 184. The issue remains left for future determination: see *Wilkes*, supra, note 144, §§135–37; *Gee*, supra, note 143, §§179–86.

170 See *Ide v ATB Sales Limited* [2008] EWCA Civ 424 (CA) §§4–6.

171 See CDC views on transmission: <https://www.cdc.gov/coronavirus/2019-ncov/prevent-getting-sick/how-covid-spreads.html?CDC_AA_refVal=https%3A%2F%2Fwww.cdc.gov%2Fcoronavirus%2F2019-ncov%2Fprepare%2Ftransmission.html>.

172 See Neil Ferguson’s evidence to the Science and Technology Parliamentary Committee on 25 March 2020 <https://parliamentlive.tv/Event/Index/2b1c71d4-bdf4-44f1-98fe-1563e67060ee> suggesting that maybe 67% of deaths were of people who would probably have died reasonably soon anyway. See also J Wise, “Covid-19: Death rate in England and Wales reaches record high because of Covid-19” (2020) 369 BMJ <https://doi.org/10.1136/bmj.m1484> and D Spiegelhalter, “How much ‘normal’ risk does Covid represent?” <https://medium.com/wintoncentre/how-much-normal-risk-does-covid-represent-4539118e1196>.

173 As to early recognition and intervention, see Q Sun et al, “Lower mortality of COVID-19 by early recognition and intervention: experience from Jiangsu Province” (2020) 10 Annals of Intensive Care 33 <https://doi.org/10.1186/s13613-020-00650-2>; see also S Begley, “With ventilators running out, doctors say the machines are overused for Covid-19” <https://www.statnews.com/2020/04/08/doctors-say-ventilators-overused-for-covid-19>.

174 But see G Lawton, “Can you catch the coronavirus twice? We don’t know yet”, New Scientist, 25 March 2020 <https://www.newscientist.com/article/mg24532754-600-can-you-catch-the-coronavirus-twice-we-dont-know-yet>; A Wajnberg et al, “Humoral immune response and prolonged PCR positivity in a cohort of 1343 SARS-CoV-2 patients in the New York City region” (2020) medRxiv <https://doi.org/10.1101/2020.04.30.20085613>.

175 See Art 15(1)(b) PLD. Five Member States have derogated from the defence. Finland and Luxembourg have not implemented it at all. Hungary excludes its application to pharmaceutical products. In Spain, all medicinal products, foods or foodstuffs intended for human consumption are excluded. Finally, in France, it does not apply to products of the human body. Furthermore, in Germany, medicines are governed by the Pharmaceutical Act 1976 (a pre-existing liability regime) that imposes liability where drugs have harmful effects that go beyond a measure defensible according to the findings of medical science.

176 See eg *Veedfald*, supra, note 127, §15; *O’Byrne v Sanofi Pasteur MSD Limited (Case C-127/04)* [2006] 2 CMLR 24 (CJEU) §25.

177 N Terry, “State of the Art Evidence: From Logical Construct to Judicial Retrenchment” (1991) 20 Anglo-American Law Review 285.

178 *Commission v United Kingdom*, supra, note 148, For commentary, see C Hodges, “Development Risks: Unanswered Questions” (1998) 61 Modern Law Review 560; M Mildred and G Howells, “Comment on ‘Development Risks: Unanswered Questions’” (1998) 61 Modern Law Review 570; J Stapleton, “Products Liability in the United Kingdom: The Myths of Reform” (1999) 34 Texas International Law Review 45; C Pugh and M Pilgerstorfer, “The Development Risks Defence – Knowledge, Discoverability and Creative Leaps” (2004) Journal of Personal Injury Law 258.

179 Section 4(1)(e) CPA.

180 *Commission v United Kingdom*, supra, note 148, §26.

181 ibid, AG§19.

182 ibid, AG§22.

183 ibid, §26.

184 ibid, §28.

185 ibid, AG§20.

186 *Sparkling Water Bottle II* (Urteil vom 9.5.1995 VI ZR 158/94 (Hamm)) NJW 1995, 2162 (BGH).

187 *A v National Blood*, supra, note 118, §77.

188 Stapleton, supra, note 178.

189 *Commission v United Kingdom*, supra, note 148, AG§21.

190 ibid, AG§20.

191 ibid, AG§§23–24.

192 *A v National Blood*, supra, note 118, §24.

193 ibid, §49.

194 Stapleton, supra, note 178, at 59.

195 *Commission v United Kingdom*, supra, note 148, AG§7.

196 Pugh and Pilgerstorfer, supra, note 178.

197 See generally R Goldberg, *Medicinal Product Liability and Regulation* (Oxford, Hart 2013), ch 7.

198 *Wilkes*, supra, note 144, §101. It was also noted that “where every aspect of the product’s design, manufacture and marketing has been the subject of the substantial scrutiny, by a regulatory authority comprised of individuals selected for their experience and expertise in the product including its safety, on the basis of full information, and that body has assessed that the level of safety is acceptable, then it may be challenging for a claimant to prove that the level of safety that persons generally are entitled to expect is at a higher level” (§100).

199 D Fairgrieve and R Goldberg, *Product Liability* (Oxford, 3^rd^ edn, Oxford University Press 2020) §§10.30, 10.43 and 11.42.

200 *Gee*, supra, note 143, §175. Andrew J did not, however, accept that “compliance with standards or regulations affords a defence or creates any *prima facie* presumption in favour of the producer” (§173).

201 ibid, §176.

202 See also M Mildred, “Pharmaceutical Products: The Relationship between Regulatory Approval and the Existence of a Defect” (2007) 6 European Business Law Review 1267, 1280, who advocates the relevance of the regulatory decision as evidence, whilst allowing the opportunity for the parties to comment upon or contradict such evidence.

203 See *Gee*, supra, note 143, §173. Such a defence has on occasion been mooted in the European discussions (see eg European Commission, *Third Report on European Product Liability Directive* (September, 2006: COM(2006) 496 final) p 11), but it has never really chimed with current English law thinking, which distinguishes between the preventative (regulation) and the *ex post facto* (liability). From a policy perspective, it would place regulatory agencies in a very difficult position, as their regulatory decisions would result in the conferral of quasi-immunities to producers in product claims.

204 See *Electricity* (Urteil vom 25.2.2014 VI ZR 144/13) (BGH) §8.

205 As M Mildred has elegantly put it: “for this defence to work, the defect must owe its existence to a regulatory requirement, a regulatory compliance defence would absolve a defendant simply because the *ex hypothesi* defective product had obtained regulatory approval”: Mildred, supra, note 202, at 1271.

206 Note, however, the recent French case, where an attempt was made to claim that the defence might apply when the information accompanying medicines – imposed by the French regulator – constituted the defect (Cass 1^er^, 27 Nov 2019, N° 18-16537, and commentary of J-S Borghetti, Revue des Contrats 2020, N°116t3, p 10 (question not resolved by the French regulator but sent back to Court of Appeal for a decision)).

207 Most notably in the USA, the Defense Production Act of 1950 (64 Stat. 798, 50 USC ch 55) has been invoked to allow the executive to direct industrial production and reportedly been used in respect of the manufacture of ventilators.

208 Section 4(1)(a) CPA.

209 There are reports that during the H1NA pandemic, vaccine producers tried to negotiate a *total* immunity from personal injury claims deriving from the vaccine, but that European countries, presenting a united front, managed successfully to resist those demands (which would probably have in any case been in breach of the PLD): see F Thoreau, C Cheneviere and N Rossignol, *Action Publique et Responsabilité Gouvernementale: la gestion de la Grippe A(H1N1) en 2009*, Courrier Hebdomadaire du CRISP, 2012/13 N° 2138-2139, p 8.

210 See generally D Fairgrieve and D Squires, *The Negligence Liability of Public Authorities* (Oxford, 2^nd^ edn, Oxford University Press 2019) at paras 5.17–5.19.

211 *Robinson v Chief Constable of West Yorkshire* [2018] AC 736 (SC) at §75.

212 *Watt v Hertfordshire County Council* [1954] 1 WLR 835 (CA).

213 Compensation Act 2006 and Social Action, Responsibility and Heroism Act 2015. See generally R Mulheron, “Legislating Dangerously: Bad Samaritans, Good Society, and the Heroism Act 2015” (2017) 80 Modern Law Review *88;* J Goudkamp, “Restating the common law? The Social Action, Responsibility and Heroism Act 2015” (2017) 37 Legal Studies 577.

214 See generally Fairgrieve and Squires, supra, note 210, §§5.20ff.

215 See Recital 2 PLD. For application of this principle as an interpretative principle of the PLD, see *Boston Scientific*, supra, note 137, §42, AG§30.

216 P Taylor, “We’re All in This Together: Extending Sovereign Immunity to Encourage Private Parties to Reduce Public Risk” (2007) 75 University of Cincinnati Law Review 1595.

217 This possibility is suggested in Thomson et al, supra, note 109.

218 In the UK, the Government is indemnifying designers and contract manufacturers of rapidly manufactured ventilator systems in respect of claims for breach of intellectual property rights and product liability: see letter of 3 April 2020 from Michael Gove MP to Public Accounts Parliamentary Select Committee, which recognises the contingent liability as being potentially “in excess of £3 million”.

219 See Fairgrieve and Goldberg, supra, note 199, ch 6.

220 Assemblée parlementaire du Conseil de l’Europe, *La gestion de la pandémie H1N1: nécessité de plus de transparence. Rapport de la Commission des questions sociales, de la santé et de la famille*, Document 12283, point 66, 7 juin 2010. See also the report of the European Parliament, *Rapport sur l’évaluation de la gestion en 2009-2010 de la grippe H1N1 en Europe*, 8 mars 2011.

221 Assemblée parlementaire du Conseil de l’Europe, *Gestion de la pandémie H1N1: nécessité de plus de transparence*, Résolution 1749 (24 juin 2010) §8.6.

222 ibid, §2.

223 Public Readiness and Emergency Preparedness Act (PREP) (2005) 42 USC §247d-6d.

224 See Notice of Declaration under the PREP Act for medical countermeasures against COVID-19 (17 March 2020) 85 FR 15198.

225 Sec 6005, H.R. 6201 (116^th^ Congress (2109-2020)).

226 Taylor, supra, note 216. One critical comment on the immunities concerned the potential leeway for discretion in pandemic emergencies to give rise to discrimination: NB Hobbes, “Out of the Frying Pan into the Fire: Heightened Discrimination & Reduced Legal Safeguards When Pandemic Strikes” (2011) 72 University of Pittsburgh Law Review 779. This seems more related to access to products than the safety of products *per se*.

227 There is also the thorny question of the funding of the scheme, whether that be global or even product-based, as there are certainly arguments in favour of producers funding schemes where injuries/side effects are due to products (especially if, for example, vaccines are produced for profit).

228 G Howells, “Justifications for Preferential Adoption of No-fault Accident Compensation Schemes”, Otago Law Review, forthcoming.

229 *Vaccine Damage I* (Urteil vom 19.2.1953 III ZR 208/51) NJW 1953 857 (BGH).

230 BGBl 1961 I, 1012. P Rott, “Compensation for Vaccination Damage under German Social Security Law”, Otago Law Review, forthcoming.

231 “Help for Victims of Immunizations” (1973) 1(5856) BMJ 758 at 759.

232 Cited in G Millward, “A Disability Act? The Vaccine Damage Payments Act 1979 and the British Government’s Response to the Pertussis Vaccine Scare” (2016) 30(2) Social History of Medicine 429 at fn 79. Lord Pearson, *Royal Commission on Civil Liability and Compensation for Personal Injury* (Cmnd 7054, 1978) vol 1 at §1398, favoured providing compensation.

233 Loi no 64-643 of 1 July 1964. It is now regulated by Art L 3111-9 Code de la Santé Publique.

234 *Office national d’indemnisation des accidents médicaux, des affections iatrogènes et des infections nosocomiales*. See generally E Rajneri, J-S Borghetti, D Fairgrieve and P Rott, “Remedies for Damage Caused by Vaccines: A Comparative Study of Four European Legal Systems” (2018) 1 European Review of Private Law 57.

235 There exists in France a sharp divide between private and public law. Substantive law normally varies according to whether the defendant is a private or a public person. In addition, civil courts, which have jurisdiction in private law matters, are distinct from administrative courts, which deal with questions pertaining to public law. ONIAM is a public person, and any claim against it must therefore be brought before an administrative court.

236 Fondazione Roselli, *Analysis of the Economic Impact of the Development Risk Clause as provided by Directive 85/374/EEC on Liability for Defective Products* (2004).

237 See generally S Taylor, *Medical Accident Liability and Redress in English and French Law* (Cambridge, Cambridge University Press 2015) ch 2.

238 See HRSA, Data and Statistics, available at <https://www.hrsa.gov/sites/default/files/hrsa/vaccine-compensation/data/monthly-stats-february-2019.pdf>. Admittedly, this is under a different damages regime where medical expenses recovery is more significant than in Europe.

239 Rott, supra, note 230.

240 *Secretary of State for Work and Pensions v G* [2017] 1 WLR 1956.

241 See generally Rajneri et al, supra, note 234, at 90.

242 We consider this most likely for vaccines, but any other pandemic product could be included.

243 See eg Fondazione Roselli, supra, note 236.

